# Metal–Organic Frameworks as Programable Platforms for Micro‐ and Optoelectronics: From Dielectrics to Semiconductors and White‐Light Emitters

**DOI:** 10.1002/smsc.70309

**Published:** 2026-06-02

**Authors:** Pounraj Thanasekaran, Arif I. Inamdar, Muhammad Usman, Ming‐Hsi Chiang, Yen‐Hsiang Liu, Kuang‐Lieh Lu

**Affiliations:** ^1^ Department of Chemistry Fu Jen Catholic University New Taipei City Taiwan; ^2^ Department of Chemistry Pondicherry University Puducherry India; ^3^ Institute of Chemistry Academia Sinica Taipei Taiwan; ^4^ Department of Medicinal and Applied Chemistry Kaohsiung Medical University Kaohsiung Taiwan

**Keywords:** dielectric, inorganic chemistry, materials science, microelectronics, modular design, nanotechnology, scalability, semiconductor

## Abstract

Metal–organic frameworks (MOFs) are emerging as highly tunable materials for microelectronic and optoelectronic applications due to their modular structures and chemically programable properties. Here we summarize recent advances in MOFs as functional electronic materials, including their use as low‐ and high‐dielectric‐constant insulators, semiconductors with enhanced charge transport, and intrinsic broadband white‐light emitters. Key strategies for bandgap and property modulation, such as metal‐node selection, π‐conjugated linker design, guest incorporation, and the role of coordinated or confined water, are critically discussed. Recent progress in electrically driven, phosphor‐free white‐light‐emitting MOFs, especially single‐component systems, is highlighted as a promising route toward simplified device architectures and environmentally sustainable alternatives to conventional luminescent materials. Remaining challenges in electrical conductivity, operational stability, and scalable device integration are assessed, alongside emerging solutions involving framework dimensionality control, improved charge transport pathways, and predictive computational modeling. Overall, MOFs are positioned as promising candidates for next‐generation nanoelectronic and optoelectronic technologies.

## Graphical abstract

Programable metal–organic framework (MOF) architectures are revealed as versatile micro‐ and optoelectronic platforms, spanning low/high‐κ dielectrics, semiconductive frameworks, and electrically driven phosphor‐free white emitters. Metal nodes, π‐conjugated linkers, guests, and confined water emerge as decisive handles for tuning charge transport, dielectric response, and sustainable light emission.

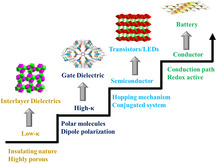

## 1 Introduction

Self‐assembled metal–organic frameworks (MOFs) are a versatile class of crystalline porous materials constructed through the coordination of organic linkers with metal ions or clusters. Over the past two decades, MOFs have attracted considerable research interest due to their exceptional attributes, including structural diversity, high surface area, tunable and uniform pore sizes, thermal and chemical robustness, functionalizable internal surfaces, high crystallinity, and intrinsic luminescence [1, 2, 3, 4, 5, 6, 7]. These unique characteristics have enabled a broad spectrum of potential applications, ranging from gas storage and separation to catalysis, sensing, magnetism, bioimaging, and drug delivery. Among these, electrical conductivity stands out as one of the earliest and most compelling properties investigated in MOFs. Nevertheless, the generally low intrinsic electrical conductivity of many MOFs has constrained their practical deployment in industrial settings.

Despite the limitation, the distinctive modularity and tunability of MOFs continue to inspire the development of advanced functional materials for emerging technologies in microelectronics and optoelectronics. In particular, the increasing demand for advanced energy storage and related technologies has prompted interest in alternative materials that potentially can substitute conventional inorganic semiconductors. MOFs offer several key advantages, including the ability to finely modulate bandgaps via structural design and the potential to combine multiple physical properties within a single framework [7, 8, 9, 10]. These features make MOFs promising candidates for integration into future‐generation electronic and optoelectronic devices. Nonetheless, while encouraging progress has been made, the application of MOFs in this domain remains in its infancy, requiring further exploration to unlock their full potential.

The construction of MOFs through advanced design strategies, such as guest molecule incorporation, composite formation, postsynthetic modification, and the development of one‐dimensional (1D) and two‐dimensional (2D) metal‐heteroatom coordination backbones (‐M‐X‐)_*n*_, has enabled precise control over both porosity and electrical conductivity. Over the past decade, a wide array of MOFs and their derivatives have been investigated for emerging applications as dielectrics, semiconductors, and conductors, as highlighted in recent review and perspective articles [8, 9, 10, 11, 12, 13, 14, 15, 16, 17, 18].

Based on their dielectric constant values, MOFs are divided into two subgroups low‐ and high‐*κ*. The controlled modulation of porosity and polarity, combined with interpenetrated topologies, has led to the realization of MOFs with tunable dielectric properties. To achieve low dielectric constants (low‐*κ*), several approaches have been employed, including the encapsulation of low‐polarizability guest species, the removal of polar molecules, and, most critically, the synthesis of highly porous, low‐density frameworks. In contrast, for high‐κ materials, the confinement of polar solvents, ion modification, and the formation of high‐density frameworks play vital roles. The presence of uniformly distributed micropores (typically < 2 nm) within MOFs aligns well with the sub‐10 nm interconnect scales in microelectronics, positioning them as ideal candidates for next‐generation dielectrics [8]. Beyond dielectric applications, the integration of semiconductive and conductive materials is vital for the advancement of microelectronic and optoelectronic devices. The development of conductive MOFs (cMOFs) necessitates a thorough understanding of charge transport mechanisms. Both experimental and theoretical investigations have revealed critical insights into the limitations of charge‐carrier mobility and pathways to improve conductivity. Three key strategies have emerged to promote intrinsic charge transport in MOFs: charge delocalization through extended covalent bonding, through‐space conduction pathways, and redox‐mediated charge hopping. These approaches have been increasingly implemented to enhance electronic performance in MOF‐based systems [10, 11, 12, 13, 14].

In parallel, MOFs have garnered growing interest as potential emitters in white light‐emitting diode (WLED) technologies, driven by the urgent need for sustainable lighting alternatives to conventional incandescent sources. MOFs offer a distinct advantage due to their multicomponent light‐emissive centers, both at the metal nodes and within the organic linkers. Their luminescent behavior can be finely tuned via diverse mechanisms, including ligand‐centered emissions, guest‐induced luminescence, ligand‐to‐ligand charge‐transfer (LLCT), metal‐to‐ligand and ligand‐to‐metal charge‐transfer (MLCT/LMCT), or synergistic combinations thereof [19, 20, 21, 22]. These mechanisms collectively position MOFs as promising phosphor materials for single‐phase WLEDs. Nevertheless, challenges persist in designing efficient, stable, and single‐phase emitters capable of overcoming the limitations of conventional multicomponent lighting systems.

**SCHEME 1:**
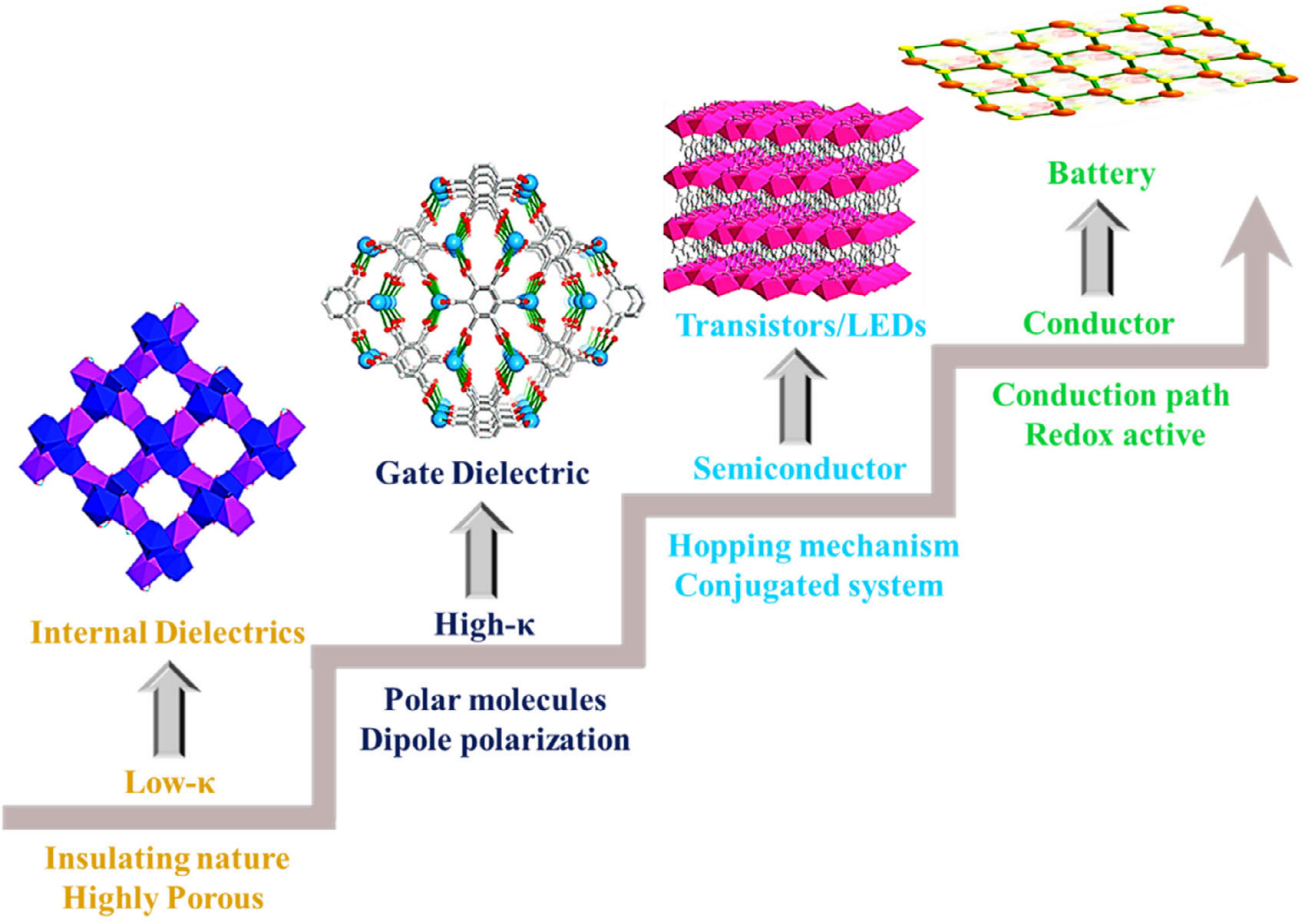
A step‐by‐step route for the development of metal–organic frameworks (MOFs).

Owing to their exceptional structural tunability and multifunctionality, MOFs enable precise modulation of electronic bandgaps spanning insulating, semiconducting, and conductive regimes. This tunability is a key determinant of whether a material exhibits dielectric or semiconducting behavior. Accordingly, this review presents a systematic overview of recent advances in the rational design of MOFs for micro‐ and optoelectronic applications, as summarized in Scheme 1.

## 2 Electronic and Optoelectronic Properties of MOFs

MOFs are widely recognized as a highly promising class of materials for energy‐related applications, owing to their tunable electronic and optical properties. These materials have demonstrated significant potential in areas such as dielectric behavior, semiconductivity, electrical conductivity, and white‐light emission. The following sections provide a detailed discussion of these functional attributes.

### 2.1 Dielectric MOFs

Dielectric materials are essential components in modern microelectronic devices, where they function as electrical insulators that respond to an applied electric field through molecular polarization. In MOFs, the dielectric constant (*κ*) can be rationally tailored by deliberate control of framework composition and structure. This section examines how intrinsic structural features of MOFs influence their dielectric response and highlights representative examples that illustrate tunable dielectric behavior.

Mendiratta et al. were among the first to demonstrate that incorporation of different anions can profoundly affect the low‐κ dielectric properties of homochiral MOFs, thereby revealing an underexplored yet effective strategy for dielectric tuning [23]. In the study, three homochiral MOFs were synthesized, {[Zn_2_(*L*‐trp)_2_(bpe)_2_(H_2_O)_2_]·2H_2_O·2NO_3_}_*n*_ (**1a**), {[Co(*L*‐trp)(bpe)(H_2_O)]·H_2_O·NO_3_}_*n*_ (**1b**), and {[Co(*L*‐trp)(bpa)(H_2_O)]·H_2_O·NO_3_}_*n*_ (**2**), where *L*‐Htrp = *L*‐tryptophan, bpe = 1,2‐bis(4‐pyridyl)ethylene, and bpa = 1,2‐bis(4‐pyridyl)ethane. These frameworks are assembled via coordination interactions between Zn^2+^ or Co^2+^ ions, bipyridyl linkers, and *L*‐tryptophan. Single‐crystal X‐ray diffraction analysis revealed that all three materials crystallize into homochiral 2D layers with a (4,4)‐connected rectangular topology and AAA stacking modes. Extensive interlayer hydrogen bonding between coordinated water molecules and nitrate guest anions was observed, playing a crucial role in stabilizing the layered architecture of **1a**.

Dielectric measurements indicated low‐κ behavior for these materials, with κ values of 2.53 for **1a** and 3.30 for **1b** at 1 MHz. In both cases, the dielectric constant decreased gradually with increasing frequency from 40 Hz to 1 MHz, consistent with the suppression of space‐charge polarization at higher frequencies. Postsynthetic anion exchange via aqueous treatment with SCN^−^, N_3_^−^, ClO_4_^−^, SO_4_^2−^, HCO_3_^−^, and H_2_PO_4_^−^ resulted in a pronounced enhancement of the dielectric constant. For the H_2_PO_4_^−^‐exchange framework (**1a**·H_2_PO_4_^−^), κ increases to 20.79 at 40 Hz, accompanied by a dielectric loss (tanδ) of 1.2. This significant enhancement was attributed to increased polarizability arising from O–H functional groups and the relatively large ionic size of the phosphate anion. These results underscore anion exchange as an effective postsynthetic strategy for modulating the dielectric properties of MOFs.

**FIGURE 1:**
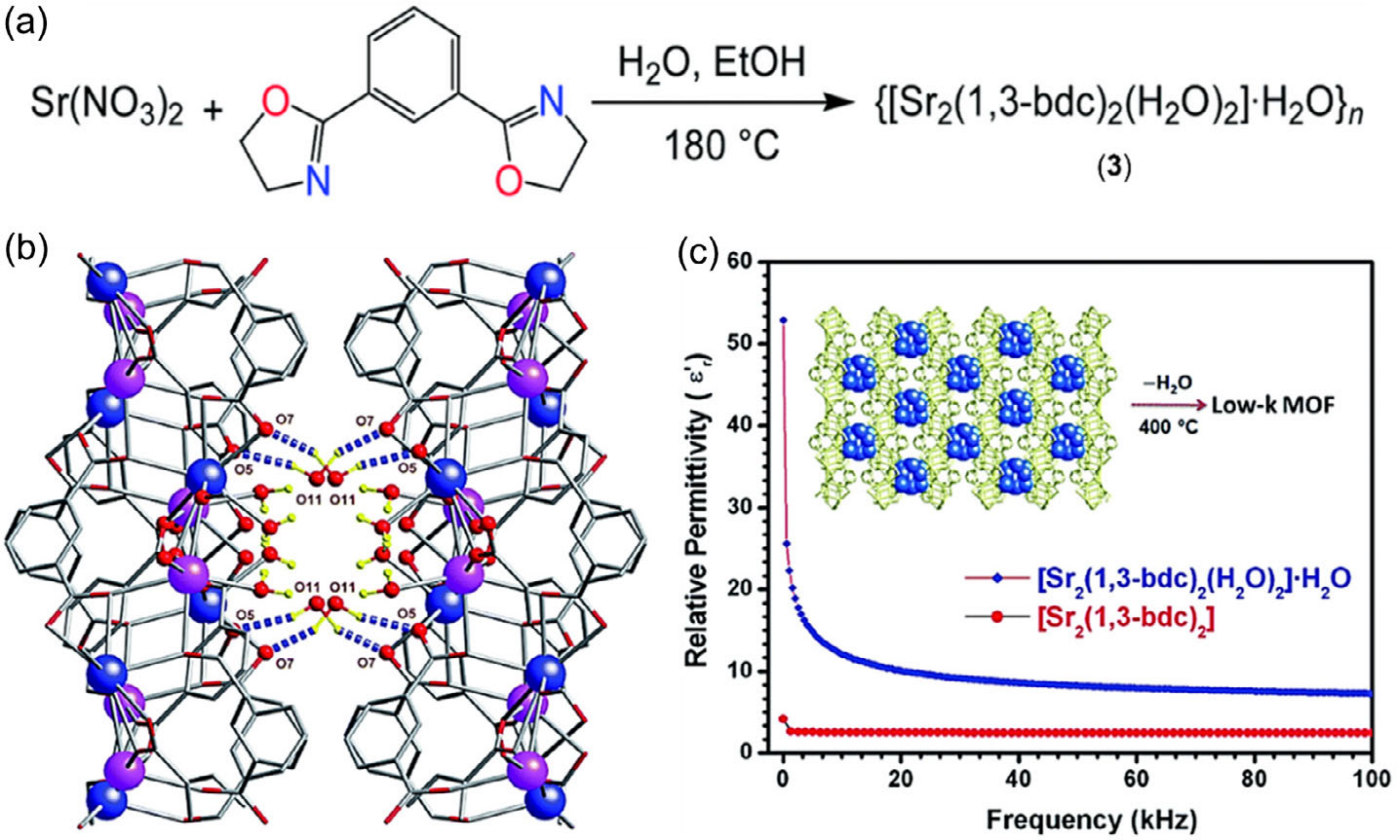
(a) One‐pot synthesis of compound **3**. (b) The host–guest hydrogen bonding interactions within **3**, and (c) relative permittivity versus frequency for **3** and the dehydrated species **3a** at room temperature. Reprinted with permission from ref. [2]. Copyright 2014, Royal Society of Chemistry.

In a related study, the first highly stable strontium‐based MOF, ([Sr_2_(1,3‐bdc)_2_(H_2_O)_2_]·H_2_O)_*n*_ (**3**), where 1,3‐bdc denotes 1,3‐bis(4,5‐dihydro‐2‐oxazolyl)benzene, was synthesized via a hydrothermal reaction (Figure 1a) [2]. Single‐crystal X‐ray diffraction analysis revealed that compound **3** adopts a 2D layered structure stabilized by strong net‐to‐net hydrogen‐bonding interactions (Figure 1b). Upon dehydration, the resulting framework [Sr_2_(1,3‐bdc)_2_] (**3a**) exhibited a markedly reduced dielectric constant.

Temperature‐dependent dielectric measurements conducted over the range of 0–350 K revealed that the hydrated phase **3** possesses a high dielectric constant of approximately 23 at 1 kHz, which is attributed to dipole polarization associated with the incorporated water molecules. In contrast, removal of these guest molecules led to a dramatic decrease in the dielectric constant to 2.4 at 0.1 MHz for the dehydrated phase **3a**, accompanied by an exceptionally low dielectric loss of 0.026 (Figure 1c). These findings highlight the critical influence of polar guest molecules and their temperature‐dependent dynamics on the dielectric response of MOF materials.

Building upon this design principle, two nickel‐based MOFs, {[Ni_2_(bbim)(H_2_bbim)_4_]·2CH_3_COO·CH_3_CN}_2_ (**4**) and (±)‐[Ni(H_2_bbim)_3_]·2Cl·2H_2_O (**5**), where H_2_bbim denotes bisbenzimidazole, were rationally synthesized to access both low‐ and high‐κ dielectric regimes through deliberate guest molecule engineering [24]. Compound **4** adopts a cyclic dimeric architecture in which weakly polar acetate and acetonitrile molecules occupy the internal voids, whereas compound **5** crystallizes as a monomeric, channel‐type framework hosting highly polar chloride anions and water molecules. This distinct contrast in guest polarity and framework topology results in markedly different dielectric responses. Under an applied electric field, compound **5** exhibits a relatively high dielectric constant, with ε_r_′(ω) reaching 12.6 at 40 Hz, which is attributed to enhanced dipolar polarization arising from the field‐induced reorientation of polar guest species. In contrast, compound **4** displays significantly lower dielectric constants of 4.76 at 40 Hz and 3.03 at 10 kHz, values that approach those of conventional low‐κ materials such as SiO_2_ (ε_r_′(ω) = 3.91 at 10 kHz). These findings clearly demonstrate the versatility of MOF platforms in spanning both high‐ and low‐dielectric regimes through synergistic modulation of framework architecture and guest composition.

Beyond transition‐metal‐based systems, the choice of metal nodes plays a pivotal role in minimizing dielectric response. Compared with strontium, magnesium, the light alkaline earth metal (group IIA), exhibits a lower refractive index and intrinsically reduced dielectric constant, making it particularly attractive for the construction of ultralow‐κ materials within porous frameworks. A hydrophobic 3D MOF, [Mg(phen)(bdc)]_*n*_ (**6**, bdc^2–^ = 1,4‐benzenedicarboxylate), was synthesized via a hydrothermal reaction [25]. Dielectric measurements revealed a gradual decrease in κ from 5.7 (at 40 Hz) to 3.3 (at 100 kHz) with increasing frequency. This frequency‐dependent behavior reflects the diminishing contributions of space‐charge and dipolar polarization at higher frequencies, leaving ionic and electronic polarization as the dominant mechanisms. Concurrently, the dielectric loss decreased from 0.2 to 0.01, which can be attributed to the suppression of interfacial polarization effects, series resistance, and electrode‐material capacitance at elevated frequencies.

**FIGURE 2:**
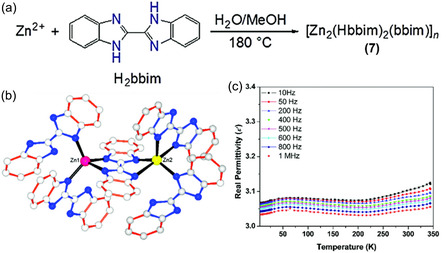
(a) One‐step synthesis of **7**. (b) Coordination environment of the Zn center. (c) Temperature‐dependent dielectric constant of **7**. Reproduced with permission from ref. [26]. Copyright 2017, Royal Society of Chemistry.

Despite these advances, guest‐free MOFs that simultaneously exhibit low dielectric constants and high thermal and solvent stability remain scarce. Addressing this challenge, Mendiratta et al*.* reported a zinc‐based MOF, [Zn_2_(Hbbim)_2_(bbim)]_*n*_ (**7**), that combines exceptional dielectric performance with robust structural integrity [26]. This framework was synthesized via a hydrothermal reaction, affording a high isolated yield of 71% (Figure 2a). Single‐crystal X‐ray diffraction analysis revealed a guest‐free, 2D honeycomb lattice with a graphene‐like AAA stacking arrangement (Figure 2b). Dielectric characterization demonstrated a consistently low dielectric constant of 3.05 at 1 MHz over a broad temperature range from 3.5 to 350 K, with minimal temperature dependence (Figure 2c). Complementary density functional theory (DFT) calculations confirmed that the observed dielectric behavior is dominated by low electronic polarizability intrinsic to the framework, underscoring the effectiveness of metal‐ligand design in achieving intrinsically low‐κ MOFs.

Table 1 compiles representative κ values for selected low‐κ MOFs, underscoring their potential as next‐generation materials for advanced dielectric applications.

**TABLE 1:** Lists of MOFs with low‐κ value (≤3.9).

MOFs	Bandgap, eV	Thermal stability, °C	Dielectric constant,κ	Dielectric loss, tanδ	Frequency, MHz	BET surface area, m^2^ g^−1^ /pore volume, cm^3^ g^−1^	Ref.
[Sr_2_(1,3‐bdc)_2_]_*n*_	n.r.	450	2.4	0.026	0.1	n.r.	[2]
{[Zn(meim)_2_]} (ZIF‐8)	n.r.	550	2.3	0.1	0.1	1947/0.663^a^	[27]
[Zn_2_(Hbbim)_2_(bbim)]_*n*_	2.91	450	3.05	0.003	1	n.r.	[26]
{[Zn_2_(*L*‐trp)_2_(bpe)_2_(H_2_O)_2_]·2H_2_O·2NO_3_}_*n*_	n.r.	223	2.5	0.01	1	n.r.	[23]
{[Co(*L*‐trp)(bpe)(H_2_O)]·H_2_O·NO_3_}_*n*_	n.r.	291	3.30	0.03	1	n.r.	[23]
[Mn_2_(D‐cam)_2_(2‐Hpao)_4_]_*n*_	n.r.	230	3.45	n.r.	0.005, 0.01, 0.1	n.r.	[28]
[Co_2_(D‐cam)_2_(3‐abpt)_2_(H_2_O)_3_]_*n*_·5*n*H_2_O	n.r.	300	2.81	n.r	0.005, 0.01, 0.1	n.r.	[28]
[Mg(phen)(bdc)]_*n*_	n.r.	260	3.3	0.2	0.1	n.r.	[25]
[Pb(Tab)_2_]_2_(PF_6_)_4_	n.r.	430	3.04	0.01	1	n.r.	[29]
[Pb(Tab)_2_(4,4^′^‐bipy)](PF_6_)	n.r.	400	2.53	0.01	1	n.r.	[29]
Ag_2_[Ag_4_Tz_6_] (FMOF‐1)	n.r.	400	1.28	n.r.	2	810.5/0.324	[30]
[Cu(FBTB)(DMF)] (FMOF‐3)	n.r.	270	2.44	n.r.	2	62/n.r.	[30]
lc‐[Cu(BTB)(DMF)]	n.r.	n.r.	2.94	n.r.	2	n.r.	[30]
ZIF‐67	n.r.	n.r.	2.39	n.r.	0.1	1780/0.73^b^	[31]
[NH_2_(CH_3_)_2_]_2_[Zn_3_(bpdc)_4_]	n.r.	360	1.80	0.005	0.1	n.r.	[32]
Al‐TCPP	n.r.	420	2.3	0.0045	10^−3^	301.957/n.r.	[33]
Basolite F300	n.r.	500	2.48	0.12	1	n.r.	[34]
MIL‐100‐MG	n.r.	n.r.	3.3	0.13	1	2055/0.993^c^	[34]
{[Ni_2_(bbim)(H_2_bbim)_4_]·2CH_3_COO·CH_3_CN}_2_	n.r.	244	3.03	0.01	10^−2^	n.r.	[24]
[Cu_3_(bdt)_2_] (HKUST‐1)	n.r.	350	2.42	0.075	1	n.r.	[35]
HKUST‐1	n.r.	n.r.	2.79	0.08	1	1007/n.r.	[36]
NEt_3_@HKUST‐1	n.r.	n.r.	2.81	0.14	1	165/n.r.	[36]

Abbreviations: 1,3‐bdc = benzene‐1,3‐dicarboxylate; 2‐Hpao = 2‐pyridinealdoxime; 3‐abpt or 3‐apt = 4‐amino‐3,5‐bis(3‐pyridyl)−1,2,4‐triazole; 4,4′‐bipy = 4,4′‐bipyridine; bdc = 1,4‐benzenedicarboxylate; bdt = benzene‐1,3,5‐tricarboxylate; CEIC = 4‐carboxy‐2‐ethyl‐1H, imidazole‐5‐carboxylate; D, cam = D(+)‐camphorate; H_2_bbim = bisbenzimidazole; H_2_FBTB = 1,4‐bis(1‐*H*‐tetrazol‐5‐yl)tetrafluorobenzene; H_2_bpdc = 4,4′‐biphenyldicarboxylic acid; HTz = 3,5‐bis(trifluoromethyl)−1,2,4‐triazole; L, trp = L, tryptophanate; bpe = 1,2‐bis(4‐pyridyl)ethylene); meim = 2‐methylimidazolate; MMA, methyl methacrylate; phen = 1,10‐phenanthroline; Tab = 4‐(trimethylammonio) benzenethiolate; TCP*P* = 4,4′, 4″’, 4″′’’‐(porphine‐5,10,15,20‐tetrayl)tetrakis(benzoic acid).

a The data is available from *Proc. Natl. Acad. Sci. U.S.A.* **2006**, *103*, 10 186–10 191. The BET surface area (1200–1950 m^2^ g^−1^) and pore volume (0.48–0.73 cm^3^ g^–1^) are varied depending on synthetic conditions.

b The data is available from *J. Chin. Chem. Soc.* **2021**, *68*, 500–506. The BET surface area (1000–1800 m^2^ g^−1^) and pore volume (0.5–0.9 cm^3^ g^–1^) are varied depending on synthetic conditions.

c The data is available from *ChemCatChem* **2017**, *9*, 971–979. The BET surface area (14–2100 m^2^ g^−1^) and pore volume (0.56–1.80 cm^3^ g^–1^) are varied depending on synthetic conditions. See *ACS Sustainable Chem. Eng.* **2020**, *8*, 8247–8255.

**FIGURE 3:**
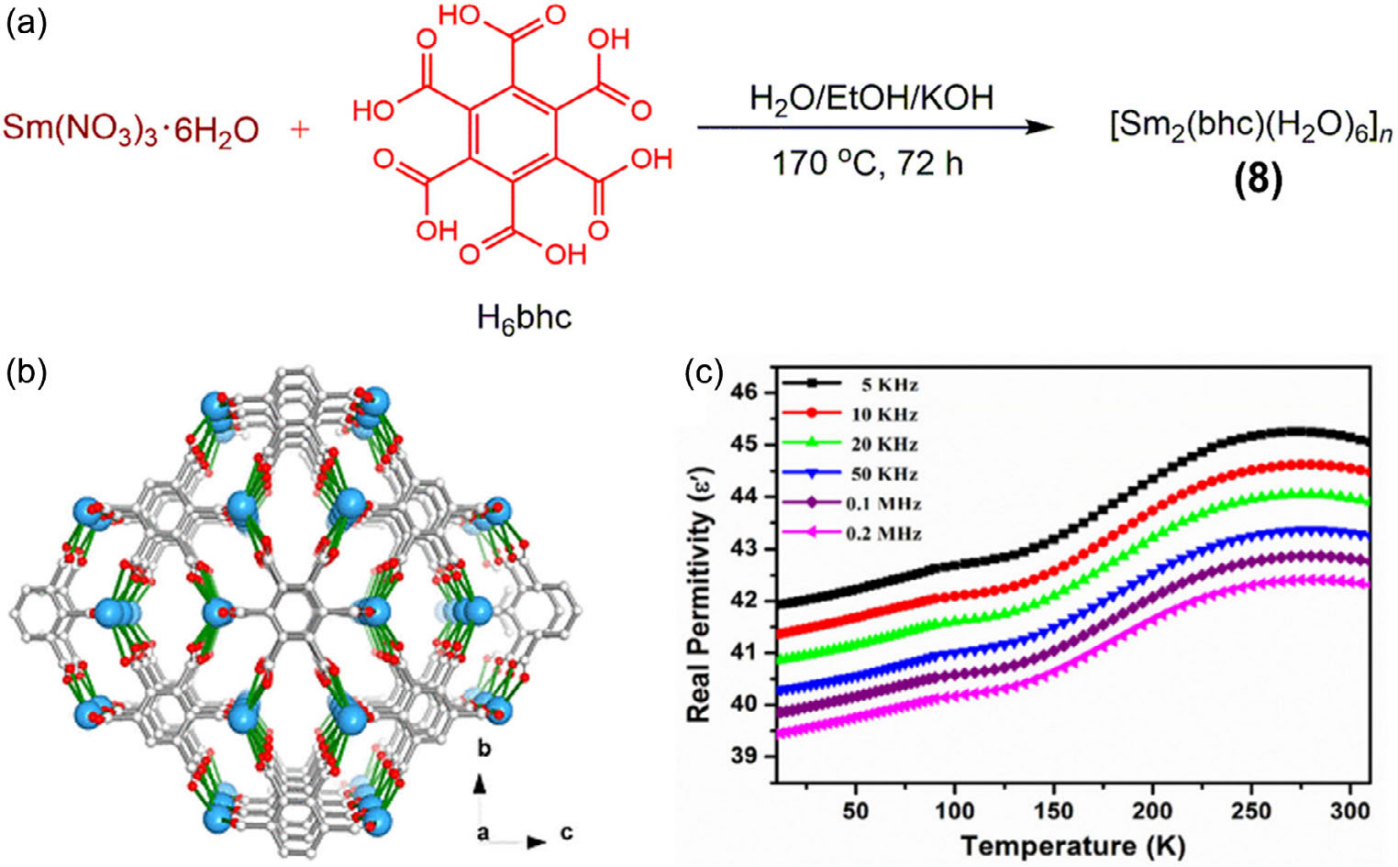
(a) Synthetic scheme of **8**. (b) Structural representation of **8** along a‐axis (Sm = blue, O = red, C = light grey). (c) Temperature‐dependent dielectric constant of **8**. Reproduced with permission from ref. [37]. Copyright 2017, American Chemical Society.

Pathak et al*.* reported a rare example of a MOF exhibiting pronounced high‐κ dielectric behavior, namely [Sm_2_(bhc)(H_2_O)_6_]_*n*_ (**8**), where bhc denotes benzenehexacarboxylate. The framework was synthesized via hydrothermal reactions (Figure 3a) [37]. Single‐crystal X‐ray diffraction analysis revealed that the hexacarboxylate ligand provides multiple coordination sites, enabling the formation of a dense, low‐porosity 3D network with a (4,8)‐connected topology (Figure 3b). This compact structural arrangement is a key contributor to the material's enhanced dielectric response.

Frequency‐dependent dielectric measurements showed that compound **8** exhibits a remarkably high relative permittivity, reaching *ε*_r_′(*ω*) = 45.1 at 5 kHz and 310 K (Figure 3c). Upon dehydration, the resulting framework [Sm_2_(bhc)]_*n*_ (**8a**) retained a relatively high dielectric constant of 27.6 at 0.2 MHz and 310 K. These values are comparable to those of conventional metal oxide gate dielectrics, such as Sm_2_O_3_, Ta_2_O_5_, HfO_2_, and ZrO_2_ [38]. The enhanced dielectric response of compound **8** arises from the synergistic contributions of coordinated water molecules, the high charge density of Sm^3+^ centers, and the intrinsically polarizable bhc ligand. Multiple polarization mechanisms, including electronic, atomic, interfacial, and ionic polarization, are particularly prominent in the low‐frequency regime. In the dehydrated phase **8a**, the high dielectric constant primarily originates from strong polarized interactions between Sm^3+^ centers and the oxygen atoms of the bhc ligand. This study represents a noteworthy example of rational MOF design aimed at high‐κ gate dielectric applications in integrated microelectronic circuits.

Complementary insights into high‐κ behavior were provided by Usman et al*.*, who investigated the influence of confined polar guest molecules on MOF dielectric properties. They reported a strontium‐based MOF, [Sr_2_(1,3‐bdc)_2_(H_2_O)(DMF)]_*n*_ (**9**, 1,3‐bdc = benzene‐1,3‐dicarboxylate) [39]. Structurally, compound **9** is closely related to the previously reported isostructural analogs [Sr_2_(1,3‐bdc)_2_(H_2_O)_2_·H_2_O]_*n*_ (**3**) and its dehydrated form [Sr_2_(1,3‐bdc)_2_]_*n*_ (**3a**). X‐ray analysis revealed that, in compound **9**, coordinated DMF molecules mediate interlayer interactions, whereas in compound **3**, water molecules serve as interlayer linkers through hydrogen bonding. Dielectric measurements performed over the temperature range of 50–300 K demonstrated that compound **9** exhibits a substantially enhanced effective dielectric constant (*κ*_eff_ = 22.4, *κ* = 39.2) at 1 MHz and 300 K, significantly exceeding those of the hydrated framework **3** (*κ*_eff_ = 7.9, *κ* = 13.0) and the dehydrated analog **3a** (*κ*_eff_ = 2.4, *κ* = 3.2). These experimental observations are in good agreement with theoretical predictions based on the Bruggeman effective medium approximation. The superior dielectric performance of compound **9** is attributed to the larger kinetic diameter (0.55 nm) and higher dipole moment (3.86 D) of DMF molecules relative to water (0.265 nm, 1.85 D) and the vacuum environment in **3a**. The presence of these highly polar guest molecules introduces additional polarization pathways, thereby amplifying the dielectric response. Collectively, these findings underscore the critical roles of both framework architecture and guest inclusion in tailoring MOF dielectric properties.

Beyond guest effects, the geometry and connectivity of organic linkers exert a decisive influence on the structural and dielectric characteristics of MOFs, particularly in the context of high‐κ materials for microelectronic applications. In a representative study, hydrothermal reactions between indium chloride and either 4,4^′^‐oxydiphthalic anhydride (odpta) or 1,2,3‐benzenetricarboxylic acid (H_3_^123^btc) yielded two structurally distinct MOFs, a flexible framework, Na[In_3_(odpt)_2_(OH)_2_(H_2_O)_2_]·(H_2_O)_4_ (**10**, odpt = 4,4^′^‐oxydiphthalate), and a rigid MOF, {[In(^123^btc)(H_2_O)_2_]·2H_2_O}_*n*_ (**11**, ^123^btc = 1,2,3‐benzenetricarboxylate) [40]. Under basic conditions, odpta undergoes hydrolysis to form a V‐shaped, eight‐coordinating oxydiphthalate linker, giving rise to the flexible MOF **10**. In contrast, the H_3_^123^btc ligand generates a rigid architecture in MOF **11** by converting its three carboxylate groups into six coordination sites. In MOF **11**, steric constraints imposed by the carboxylate linkers induce a distorted pentagonal bipyramidal coordination geometry, facilitating interactions with both coordinated and guest water molecules. Dielectric measurements conducted over the temperature range of 20–300 K revealed that MOF **11** exhibits a significantly higher dielectric constant (κ = 56.3 at 1 kHz) than MOF 10 (κ = 40.5 at 1 kHz), a difference primarily attributed to the higher content of guest water molecules in MOF **11**. Frequency‐dependent measurements (20 Hz‐1 MHz) further supported this trend, with ε_r_′(ω) values of 45.7 for MOF **11** and 32.7 for MOF **10** at 1 MHz and 300 K. The observed dielectric behavior reflects the combined effects of coordinated and guest water polarization, extended metal–oxygen chains, and framework density.

**FIGURE 4:**
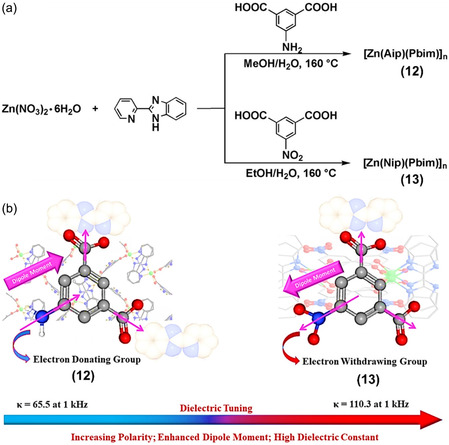
(a) One‐step synthesis of **12** and **13**. (b) Schematic of **12** and **13** showing correlation of the electron donating and withdrawing groups with dielectric constants.

The influence of linker substituents on dielectric properties was further examined through two zinc‐based MOFs incorporating electron‐donating and electron‐withdrawing functional groups, [Zn(Pbim)(Aip)]_*n*_ (**12**) and [Zn(Pbim)(Nip_3_)]_*n*_ (**13**), where Pbim = 2‐(2‐pyridyl)benzimidazole, Ai*p* = 5‐aminoisophthalate, and Ni*p* = 5‐nitroisophthalate (Figure 4a,b) [41]. Dielectric measurements of pelletized samples across varying temperatures and frequencies revealed that the dielectric constants of both materials increase with temperature at 1 kHz but decrease with increasing frequency up to 1 MHz. Remarkably high dielectric constants of κ = 65.5 for MOF **12** and κ = 110.3 for MOF **13** were observed. These elevated values are attributed to the presence of polar –NH_2_ and –NO_2_ substituents, which modify the local electronic environment through inductive effects and suppress electron conduction along the Zn–O chains. Additional contributions from space‐charge and dipolar polarization associated with these functional groups further enhance the dielectric response.

Collectively, these studies highlight the critical roles of linker geometry, functional substituents, and framework architecture in modulating the dielectric properties of MOFs. In parallel, hydrophobicity has emerged as a promising yet underexplored design strategy for dielectric materials in microelectronic applications, offering the potential to improve long‐term stability and device reliability by mitigating moisture‐induced degradation processes such as corrosion, swelling, and metal oxidation. Incorporating hydrophobic features may therefore enhance both thermal and environmental resilience.

**FIGURE 5:**
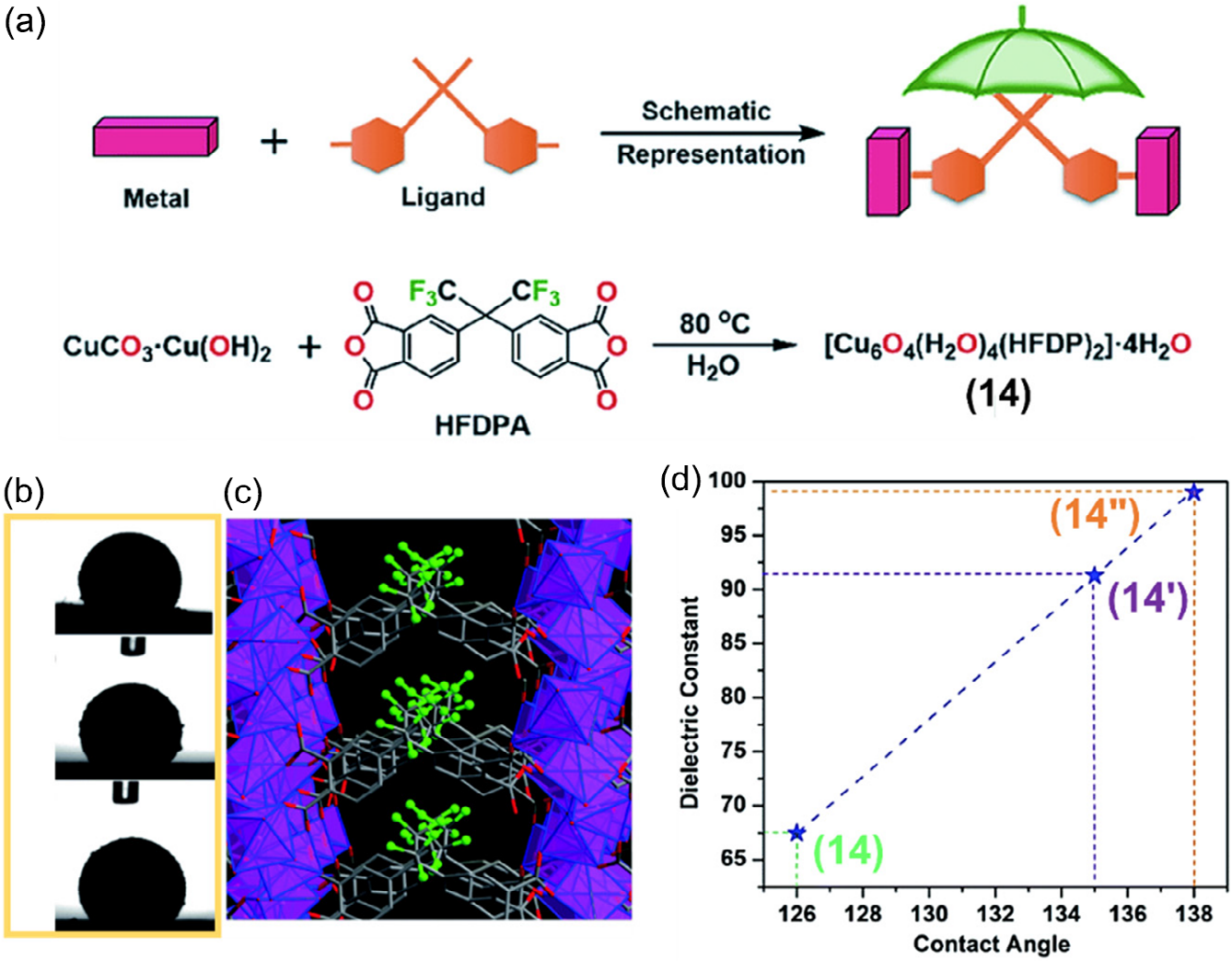
(a) Schematic representation of the environmentally friendly synthesis of self‐protecting hydrophobic Cu–MOF umbrella. (b) Contact angle measurements of **14** (126°), **14’** (135°), and **14”** (138°). (c) The crystal structure of **14** demonstrates the outward direction of the fluorocarbon groups. (d) The linear relationship between the dielectric constant and contact angles for **14**, **14’,** and **14”**. Reproduced with permission from ref. [3]. Copyright 2020, Royal Society of Chemistry.

In this context, Inamdar et al*.* reported the synthesis of a self‐protecting hydrophobic copper‐based MOF, [Cu_6_O_4_(H_2_O)_4_(HFDP)_2_]·4H_2_O (**14**), where HFD*P* = 4,4^′^‐(hexafluoroisopropylidene)diphthalate) [3]. Single‐crystal X‐ray diffraction analysis revealed that HFDP ligands, generated in situ through hydrolysis of 4,4^′^‐(hexafluoroisopropylidene)diphthalic anhydride, coordinate to Cu centers in a μ8 fashion (Figure 5a), forming a robust framework stabilized by extensive hydrogen‐bonding interactions involving carboxylate groups and both coordinated and guest water molecules. Despite the intrinsic hydrophilicity of carboxylate functionalities, the incorporation of perfluorinated CF_3_ groups imparts pronounced hydrophobicity, as evidenced by water contact angles of 126°, 135°, and 138° for the pristine **14**, partially desolvated **14’**, and fully desolvated **14”** forms, respectively (Figure 5b). Dielectric characterization of compound **14** revealed a high dielectric constant of 67.44 with a low dielectric loss (tanδ = 0.05) at 1 kHz and 300 K. Progressive structural transformations from **14** to its partially and fully desolvated forms, accompanied by reduced pore volume, reinforced hydrogen‐bonding networks, and modified metal–oxygen chains (Figure 5c), resulted in further increases in the dielectric constant to 91.29 and 98.99, respectively (Figure 5d). This work represents the first demonstration of a MOF that simultaneously combines high dielectric permittivity with pronounced hydrophobicity, offering a compelling strategy for the development of self‐protecting dielectric materials in next‐generation microelectronic devices.

From the preceding discussion, it is evident that the dielectric constant of MOFs is strongly correlated with their structural features, particularly framework polarity and porosity. Highly porous MOFs with large void volumes and nonpolar or weakly polar linkers generally exhibit low‐κ behavior, as the vacuum‐like pores and reduced polarizable density suppress overall polarization. Conversely, dense or less porous frameworks containing polar functional groups, electron‐rich moieties, or retained polar guest molecules promote higher κ values through enhanced orientational and electronic polarization under an applied electric field.

The inherent tunability of MOFs via rational choice of metal nodes, organic linkers, postsynthetic modification, and host–guest chemistry offers distinct advantages over conventional inorganic dielectrics, enabling precise tailoring of κ across a wide range. Key design strategies include increasing porosity and surface area or removing polar solvents for low‐κ applications, and introducing polarizable groups or minimizing voids for high‐κ behavior. This structure–property relationship provides a rational foundation for the targeted design of application‐specific dielectric MOFs.

Representative classes of high‐κ MOFs are summarized in Table 2, underscoring the novelty and importance of recent contributions in this emerging field.

**TABLE 2:** Lists of MOFs with high‐κ values (>3.9).

MOFs	Band gap, eV	Thermal stability, °C	Dielectric constant, κ	Dielectric loss, tanδ	Frequency, MHz	BET surface area, m^2^ g^−1^/ pore volume, cm^3^ g^−1^	Ref.
Cd‐MOF	n.r.	n.r.	38.6	16.0	1	n.r.	[42]
{[Zn(TBPR)⊂0.25(HClO_4_)](0.25HClO_4_)}_*n*_	n.r.	n.r.	80.0	0.4	10^−3^	n.r.	[43]
[Sr(μ‐BDC)(DMF)]_∞_	n.r.	550	19.0	0.9	10^−5^	n.r.	[44]
[Sr(μ‐BDC)]_∞_	n.r.	600	27.5	1.4	10^−5^	5.9	[44]
[Sr(TDA)(DMF)]_∞_	n.r.	225	14.0	0.01	1	n.r.	[45]
[Cu_2_(EBTC)(H_2_O)_2_·8H_2_O·DMF·DMSO]_∞_	n.r.	307	82.8	0.35	10^−5^	1852^a^/n.r.	[46]
[Cu_2_(EBTC)]_∞_	n.r.	307	6.2	0.018	10^−5^	n.r.	[46]
[NH_2_CH^+^NH_2_][Mn(HCOO)_3_] (FMDMn)	n.r.	n.r.	11.0	0.05	1.2 × 10^−5^	n.r.	[47]
[(pnH_2_^2+^)_2_(H_2_O)][Mg(HCOO)_3_]_4_	n.r.	200	37.0	0.04	1	n.r.	[48]
{[Cu(CA)]·CH_3_CN}_*n*_	n.r.	210	44.0	4.0	10^−4^	n.r.	[49]
{[Cu(CA)(H_2_O)_2_]·H_2_O}_*n*_	n.r.	410	35.0	4.5	10^−4^	n.r.	[49]
[Ag_2_(CA)]_*n*_	n.r.	300	321.0	14.0	10^−4^	n.r.	[49]
{[H_2_N(CH_3_)_2_][Zn(TBTC)]}·2DMF·EtOH	4.23	300	19.5	0.003	1	n.r.	[50]
[Sr(2‐PZC)_2_]	n.r.	415	8	0.002	1	n.r.	[51]
[Sr(2,5‐PDC)(DMF)]	n.r.	145	9.6	0.003	1	n.r.	[51]
[Sr(2,5‐PDC)(DMA)]	n.r.	180	12.6	0.005	1	n.r.	[51]
[Sr(IDA)_2_]	n.r.	150	12.0	0.005	1	n.r.	[51]
[Sr(SBA)(H_2_O)_2_]·DMF	n.r.	450	17.0	0.004	1	n.r.	[51]
[Sr_2_(FBA)_2_(H_2_O)_2_ (DMA)_2_]·DMA	n.r.	450	20.4	0.008	1	n.r.	[51]
[Sr(BDC)(DMA)(H_2_O)]	n.r.	150	14.2	0.001	1	n.r.	[52]
[Ca(ABDC)(DMF)]	n.r.	215	13.7	0.009	1	n.r.	[52]
[Ca(ABDC)(DMF)_2/3_]	n.r.	115	11.7	0.008	1	n.r.	[52]
[Sr(ABDC)(DMF)]	n.r.	400	19.2	0.005	1	n.r.	[52]
[Sr(OBA)(MF)]	n.r.	250	13.2	0.004	1	n.r.	[52]
{[Sr(FBA)(DMA)(H_2_O)]·(DMA)(EtOH)(H_2_O)}	n.r.	70	36.2	0.001	1	n.r.	[52]
[C_4_H_12_N_2_][Ag_4_(hedp)_2_]	3.71	220	16,000	10.8	2.5 × 10^−4^	n.r.	[53]
[C_4_H_12_N_2_][Ag_10_(hedp)_4_(H_2_O)_2_]·2H_2_O	3.49	220	21,000	9.2	2.5 × 10^−4^	n.r.	[53]
[Sm_2_(bhc)(H_2_O)_6_]_*n*_	n.r.	445	45.1	0.023	5 × 10^−3^	n.r.	[37]
[Sm_2_(bhc)]_*n*_	n.r.	n.r.	27.6	0.024	0.2	n.r.	[37]
[Sr_2_(1,3‐bdc)_2_(H_2_O)(DMF)]_*n*_	n.r.	570	39.2	0.065	1	10.11/n.r.	[39]
Na[In_3_(odpt)_2_(OH)_2_(H_2_O)_2_]·(H_2_O)_4_	n.r.	300	405	0.06	10^−3^	n.r.	[40]
{[In(^123^btc)(H_2_O)_2_]·2H_2_O}_*n*_	n.r.	400	56.3	0.09	10^−3^	n.r.	[40]
[Cu_6_O_4_(H_2_O)_4_(HFDP)_2_]·4H_2_O	n.r.	200	67.44	0.050	10^−3^	n.r.	[3]
[Cu_6_O_4_(H_2_O)_4_(HFDP)_2_]	n.r.	170	91.29	0.051	10^−3^	65.24/n.r.	[3]
[Cu_6_O_4_(HFDP)_2_]	n.r.	220	98.99	0.044	10^−3^	48.16/n.r.	[3]

Abbreviations: 1,3‐bdc = benzene‐1,3‐dicarboxylic acid; 2,5‐PDC = 2,5‐pyridinedicarboxylate; 2‐PZC = 2‐pyrazinecarboxylate; ^123^btc = 1,2,3‐benzenetricarboxylate; ABDC = 2‐aminoterephthalic acid, OBA = 4,4′‐oxybis(benzoic acid); BDC^2−^ = benzene‐1,4‐dicarboxylate; bhc = benzenehexacarboxylate; btc = benzene‐1,3,5‐tricarboxylate; CA = 4‐pyranone‐2,6‐dicarboxylic acid; EBTC = 1,10‐ethynebenzene‐3,3′, 5,5′‐tetracarboxylate; FBA = 4,4′‐(hexafluoroisopropylidene)bis(benzoate); H_3_TBTC = 1,3,5‐tris[4‐(carboxyphenyl)oxamethyl]−2,4,6‐trimethylbenzene; hed*p* = 1‐hydroxyethane‐1,1‐diphosphonic acid; HFDPA = 4,4′‐(hexafluoroisopropylidene)diphthalic diacetate; IDA, iminodiacetate; MF = N, methylformamide; odpt = 4,4′‐oxydiphthalate; pnH_2_^2+^ = di‐protonated 1,3‐propanediamine; SBA = 4,4′‐sulfonyldibenzoate; TBPR, (*S*)‐*N*‐2‐tetrazoylbenzylproline; TDA, thiophene‐2,5‐dicarboxylate.

a The data is available from *Chem. Commun.* **2009**, 7551−7553.

### 2.2 Semiconductive and Conductive Properties of MOFs

As discussed in the preceding sections, MOFs have been widely explored as passive dielectric materials, where low framework density and suppressed polarization yield reduced dielectric constants and leakage currents suitable for insulating layers in microelectronic devices. Beyond this role, controlled bandgap engineering enables MOFs to function as active semiconductive or conductive layers, provided that electronic transport is intrinsic to the framework rather than arising from ionic motion or defect‐mediated leakage. In this section, we examine how framework dimensionality, metal–ligand orbital coupling, and supramolecular interactions in MOFs dictate their suitability for active device integration.

**FIGURE 6:**
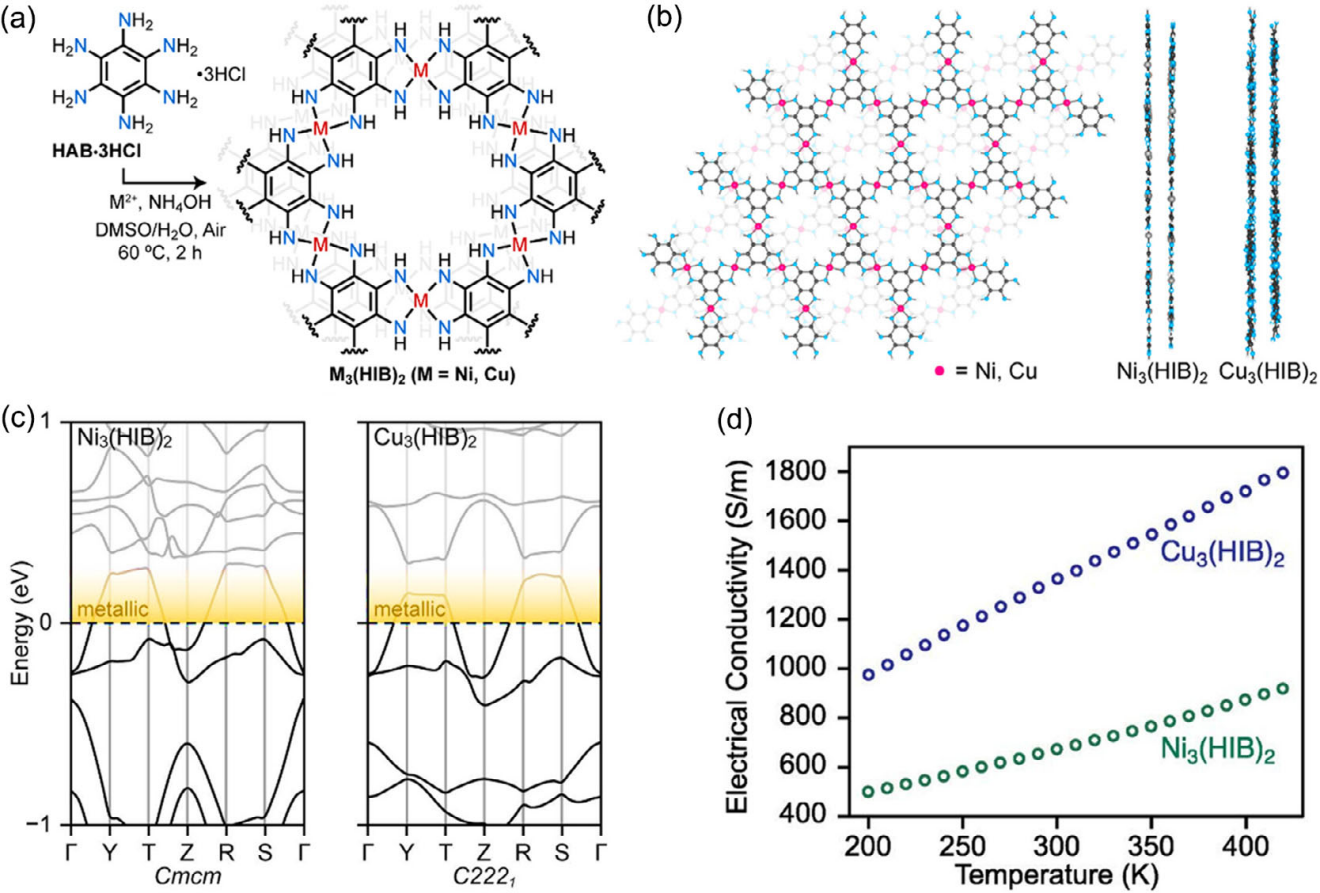
(a) Synthesis schematic of M_3_(HIB)_2_ (M = Ni, Cu) and (b) structural representation for M_3_(HIB)_2_. (c) Band structure of bulk Ni_3_(HIB)_2_ and Cu_3_(HIB)_2_. (d) Temperature‐dependent conductivity measured for pellets of M_3_(HIB)_2_ via the van der Pauw method. Reproduced with permission from ref. [54]. Copyright 2017, American Chemical Society.

2D cMOFs exemplify the highest level of electronic delocalization achieved in MOF chemistry. Ni_3_(HIB)_2_ and Cu_3_(HIB)_2_ (HIB = hexaiminobenzene) form extended π–d conjugated sheets in which metal 3d orbitals and ligand π orbitals contribute jointly to the electronic bands (Figure 6a,b) [54]. DFT calculations indicate partially filled bands with metallic‐like dispersion (Figure 6c), consistent with experimentally measured room‐temperature conductivities of 800 S m^−1^ (Ni_3_(HIB)_2_) and 1300S m^−1^ (Cu_3_(HIB)_2_) obtained from pellet samples under vacuum and dark conditions (Figure 6d). Importantly, conductivity increases linearly with temperature between 200 and 300 K, indicating thermally activated semiconductive behavior rather than ideal metallic transport. From a microelectronic perspective, these values place cMOFs within a semiconductive regime compatible with active channel layers, where controlled carrier density and temperature response are desirable rather than detrimental.

In contrast, carboxylate‐based alkaline‐earth MOFs demonstrate how electronic transport can be intentionally suppressed to reinforce dielectric functionality. Through ligand engineering and metal selection, Usman et al. demonstrated that strong coordination between Sr^2+^ ions and oxazoline‐containing ligands effectively mitigates unintended electronic conduction in the parent framework [Sr_2_(1,3‐bdc)_2_(H_2_O)_2_]·(H_2_O)_*n*_ (**3**) [2]. Dehydration yields phase **3a** with a dramatic reduction in ionic conductivity from 6.56 × 10^−6^ to 4.96 × 10^−9^ S cm^−1^, accompanied by exceptionally low leakage currents (1.69 × 10^−9^ A mm^−2^) in metal–insulator–metal (MIM) devices. The results confirm that charge transport is effectively suppressed. These characteristics firmly position compound **3a** as a low‐κ insulating layer, reinforcing continuity with the dielectric‐focused discussion in earlier sections and establishing a clear boundary between passive and active MOF functionalities.

Between these two extremes, 3D Sr‐based MOFs incorporating hydrogen‐bonding networks demonstrate how moderate, controllable electronic transport can emerge without sacrificing thermal or structural stability. The structure of [Sr(H^124^btc)(H_2_O)]_*n*_ (**15**, H_3_^124^btc = 1,2,4‐benzenetricarboxylic acid) shows that the carboxylate group of the H^124^btc^2−^ ligand participates in framework extension, while hydrogen‐bonded water molecules reinforce the layered architecture [55]. The framework exhibits remarkable thermal stability up to 520°C. The MOF **15** exhibits semiconductive behavior with conductivities in the 10^−6^ S cm^−1^ range and a low activation energy of 0.17 eV, reflecting thermally assisted charge hopping facilitated by hydrogen‐bonded pathways. The direct bandgap of 2.3 eV places this material within the range of classical inorganic semiconductors such as CdSe, ZnTe, and CdTe [56]. This work constitutes the first demonstration of a 3D Sr‐based MOF exhibiting semiconductive behavior compatible with microelectronic integration, suggesting potential utility in low‐current electronic or sensing components.

**FIGURE 7:**
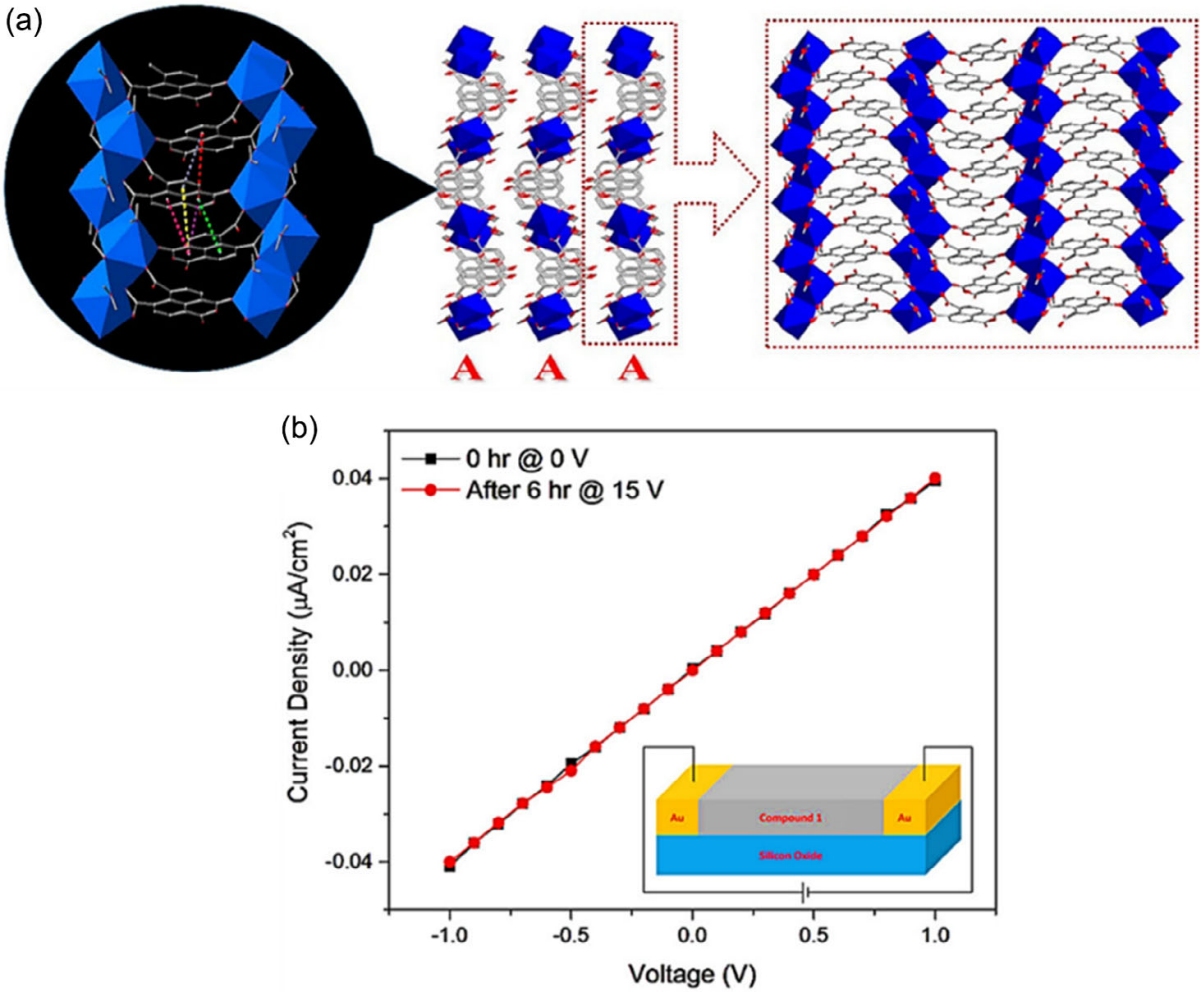
(a) 3D MOF structure of **16**, composed of an AAA arrangement, where A represents the 2D sheet (O = red and C = light gray). (b) The current–voltage (*I*−*V* ) plot of the MOF under ambient conditions before and after applying a constant voltage of 15 V for 6 h. The inset is a schematic diagram of the device used for electrochemical study. Reproduced with permission from ref. [1]. Copyright 2016, American Chemical Society.

Charge transport can be further enhanced through ligand‐centered π−π stacking interactions. In {[Sr(ntca)(H_2_O)_2_]·H_2_O}_*n*_ (**16**), where ntca = 1,4,5,8‐naphthalenetetracarboxylic acid) [1], and related benzene‐1,2,3‐tricarboxylate frameworks, {[Sr(H_2_^123^btc)_2_(MeOH)(H_2_O)_2_]·2H_2_O} (**17**) [57], face‐to‐face π−π interactions create preferential conduction pathways along stacked aromatic columns (Figure 7a). Single‐crystal current–voltage (*I*−*V* ) measurements reveal conductivities up to 10^−4^−10^−3^ S cm^−1^ (Figure 7b). The values are significant for molecular‐scale electronic elements while remaining distinct from metallic interconnect requirements. These systems demonstrate that ligand design alone can modulate conductivity over several orders of magnitude.

**FIGURE 8:**
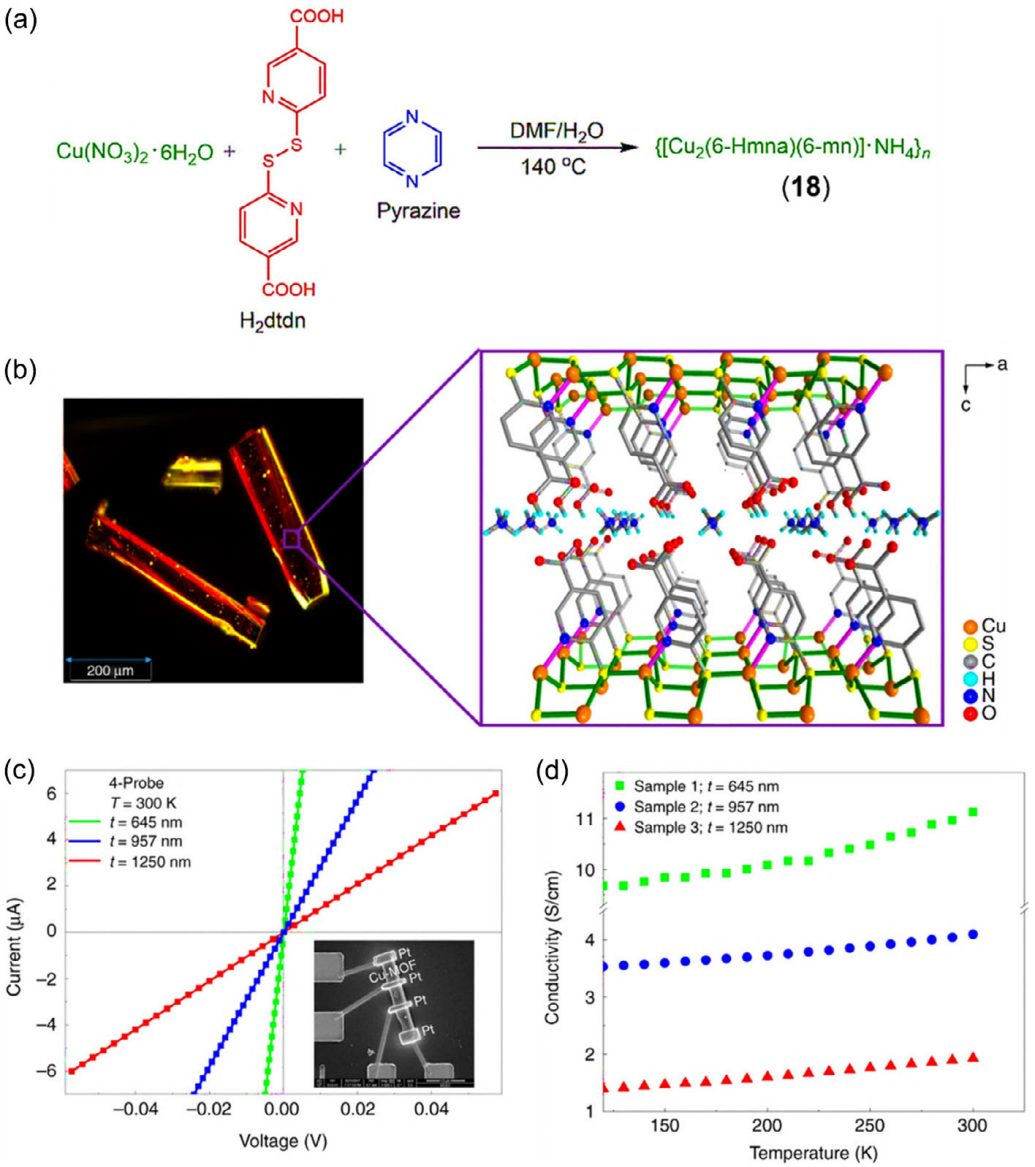
(a) Synthetic scheme of {[Cu_2_(6‐Hmna)(6‐mn)]·NH_4_}_*n*_ (**18**). (b) Dark‐field optical image and the structure of **18** (Cu = orange, O = red, C = light gray, N = blue, S = yellow, H = cyan). (c) Four probe *I*–*V* measurements of **18** (Inset figure represents the actual device). (d) Temperature‐dependent conductivity of **18** at 0.1 V obtained via the four‐probe method*.* Reproduced from ref. [4]. Available under a CC‐BY 4.0 license. Copyright 2019, Springer Nature.

A major leap in electrical performance is achieved when metal–sulfur coordination motifs extend from 1D chains into 2D planes. The Cu–S framework {[Cu_2_(6‐Hmna)(6‐mn)]·NH_4_}_*n*_ (**18**, 6‐Hmna = 6‐mercaptonicotinic acid, 6‐mn = 6‐mercaptonicotinate) exhibits a honeycomb‐like (–Cu–S–)_*n*_ sheet structure (Figure 8a,b) with pendant ligands forming a porous layered architecture stabilized by hydrogen bonding with NH_4_^+^ ions [4]. Strong Cu 3d–S 3p orbital overlap yields single‐crystal conductivities as high as 10.96 S cm^−1^, with thickness‐dependent variations attributed to defect density and contact resistance (Figure 8c). Combined with a narrow band gap (1.34 eV) and ultralow activation energy (6 meV), compound **18** functions as a true semiconductive active layer, approaching the performance of conventional inorganic semiconductors while retaining the synthetic tunability of MOFs. This represents a critical benchmark for MOF integration into microelectronic device architectures requiring efficient in‐plane charge transport.

**FIGURE 9:**
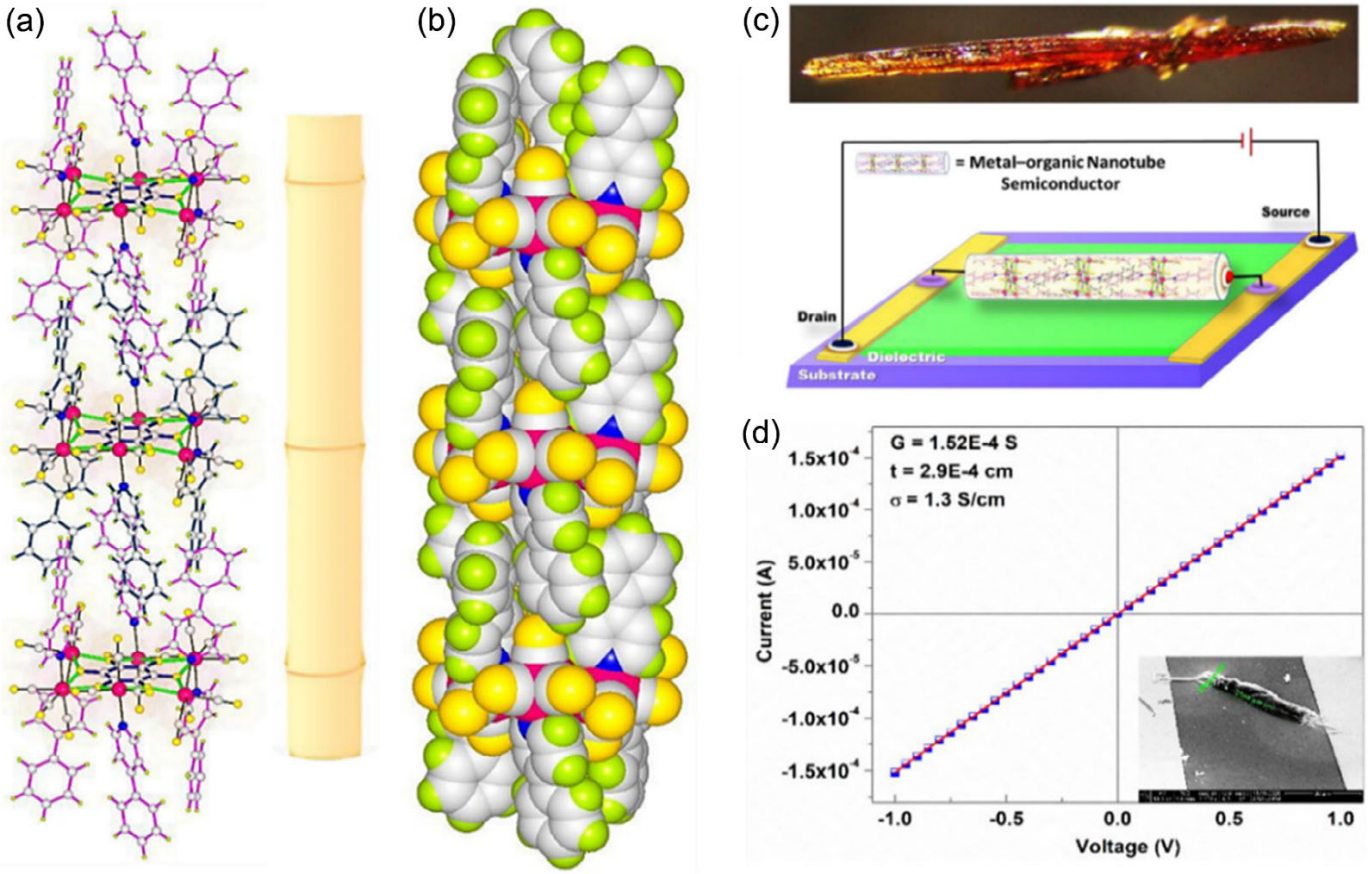
Structural representation of compound **19b**. (a) Bamboo‐like topology of compound **19b**. (b) Space‐fill structural representation of the molecular bamboo culm **19b**. (c) Actual image of the **19b** single crystal and a schematic diagram of the device used for conductivity measurements. (d) *I*−*V* characterization curve of **19b** (SEM image of the actual device appears in the inset). Reproduced with permission from ref. [59]. Copyright 2022, American Chemical Society.

Metal–organic nanotubes (MONTs) further expand the design space for active MOF electronics by combining 1D confinement with tunable π‐conjugation. Rhenium‐based MONTs [{Re(CO)_3_}_6_(C_6_O_6_)(Py‐R)_6_]_*n*_, where C_6_O_6_^6−^ = benzene‐1,2,3,4,5,6‐hexaolate (bho^6−^) linker, and Py‐R represents pyridine‐based ligands with varying substituents (R = CH_3_CO (**19a**); C_6_H_5_ (**19b**); H (**19c**)) [58, 59, 60], exhibit bamboo‐like architectures, where the pyridyl segments act as periodic internodes separated by approximately 2 nm (Figure 9a,b). The tubular structure effectively facilitates through‐bond and through‐space charge transport, supported by a low activation energy (1.63 and 8.4 meV for **19b** and **19c**, respectively). Among them, compound **19b** exhibits an optical bandgap of approximately 1.03 eV, consistent with DFT predictions (1.2 eV) [59]. Comparative analysis on calculated electronic structures of MONTs reveal that increasing linker conjugation raises the HOMO level and effectively reduces the HOMO−LUMO gap, facilitating charge transport. These data establish a rational strategy for tuning MONT electronic properties.

*I*−*V* measurements revealed that AC conductivity of compound **19b** increased with frequency due to an electron‐hopping mechanism. Notably, single‐crystal devices show conductivities up to 1.5 S cm^−1^ (Figure 9c,d), four orders of magnitude higher than pelletized samples (~10^–4^ S cm^−1^), highlighting the importance of crystallinity and grain‐boundary suppression for device‐relevant performance. Extension of π‐conjugation in compound **19c** further narrows the bandgap and enhances conductivity to 2.4 S cm^−1^. These results demonstrate that MONTs can be rationally engineered as active transport elements, distinct from porous dielectric MOFs discussed earlier.

**FIGURE 10:**
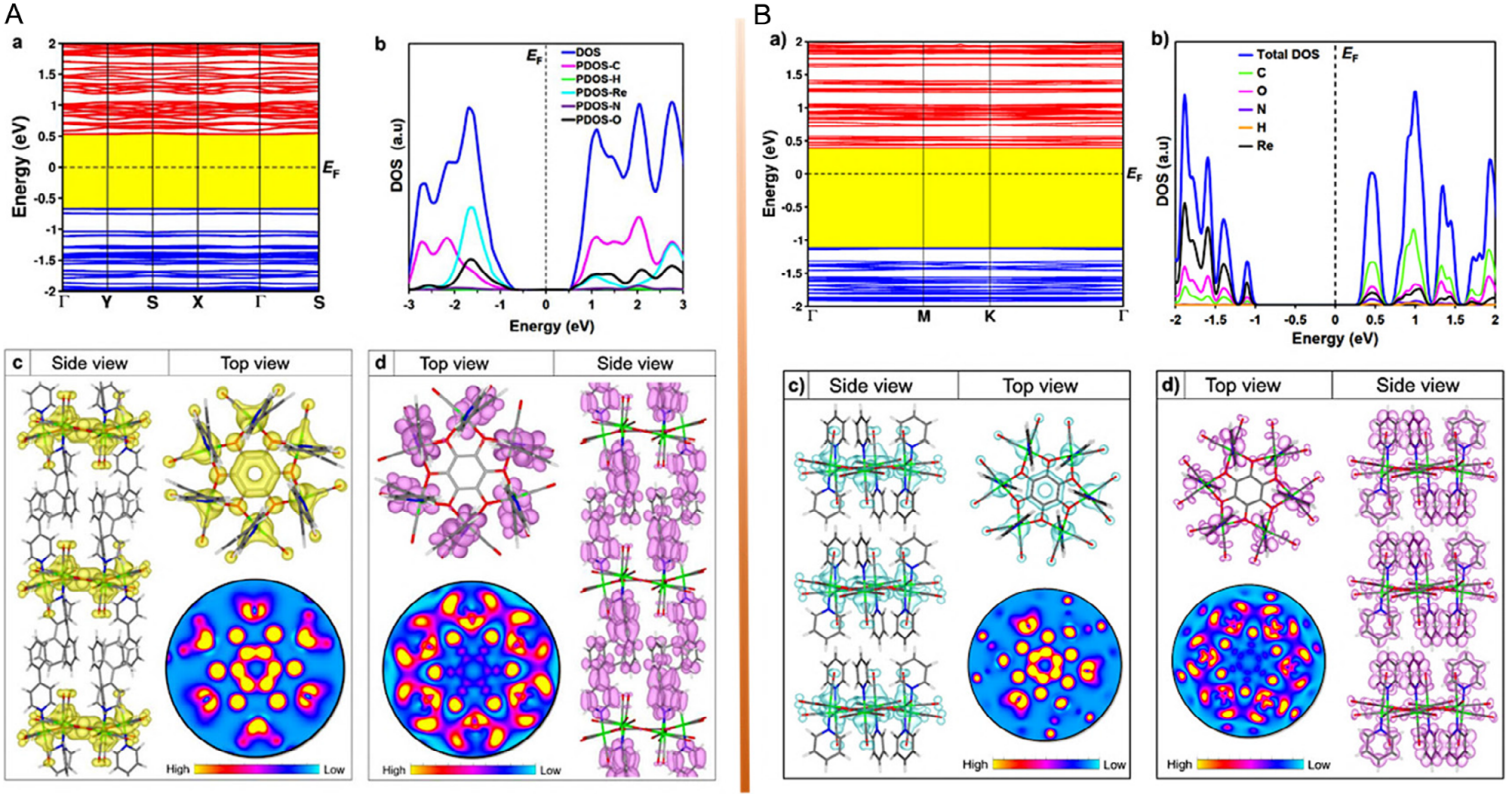
Theoretical bandgap presentation of **19b** (left panel) and **19c** (right panel). (a) Calculated band structure, (b) density of states (DOS) and projected DOS (PDOS) plots, and (c) valence bond maximum (VBM) and (d) conduction bond maximum (CBM). Reproduced with permission from refs. [59, 60]. Copyright 2022 and 2025, American Chemical Society.

Compound **19b** also exhibits pronounced photoconductivity, with a photoconductive gain of 744 A W^−1^ and responsivity of 243 A W^−1^ under 405 nm illumination, with carrier lifetimes ranging from 4.5 to 9.6 s. DFT calculations suggest that spatial separation of electrons and holes within the nanotube promotes long carrier lifetimes and efficient charge transport (Figure 10). The pronounced photoconductivity observed under visible excitation further positions MONTs as promising candidates for optoelectronic and photodetector applications, where combined electrical and optical functionality is required.

**FIGURE 11:**
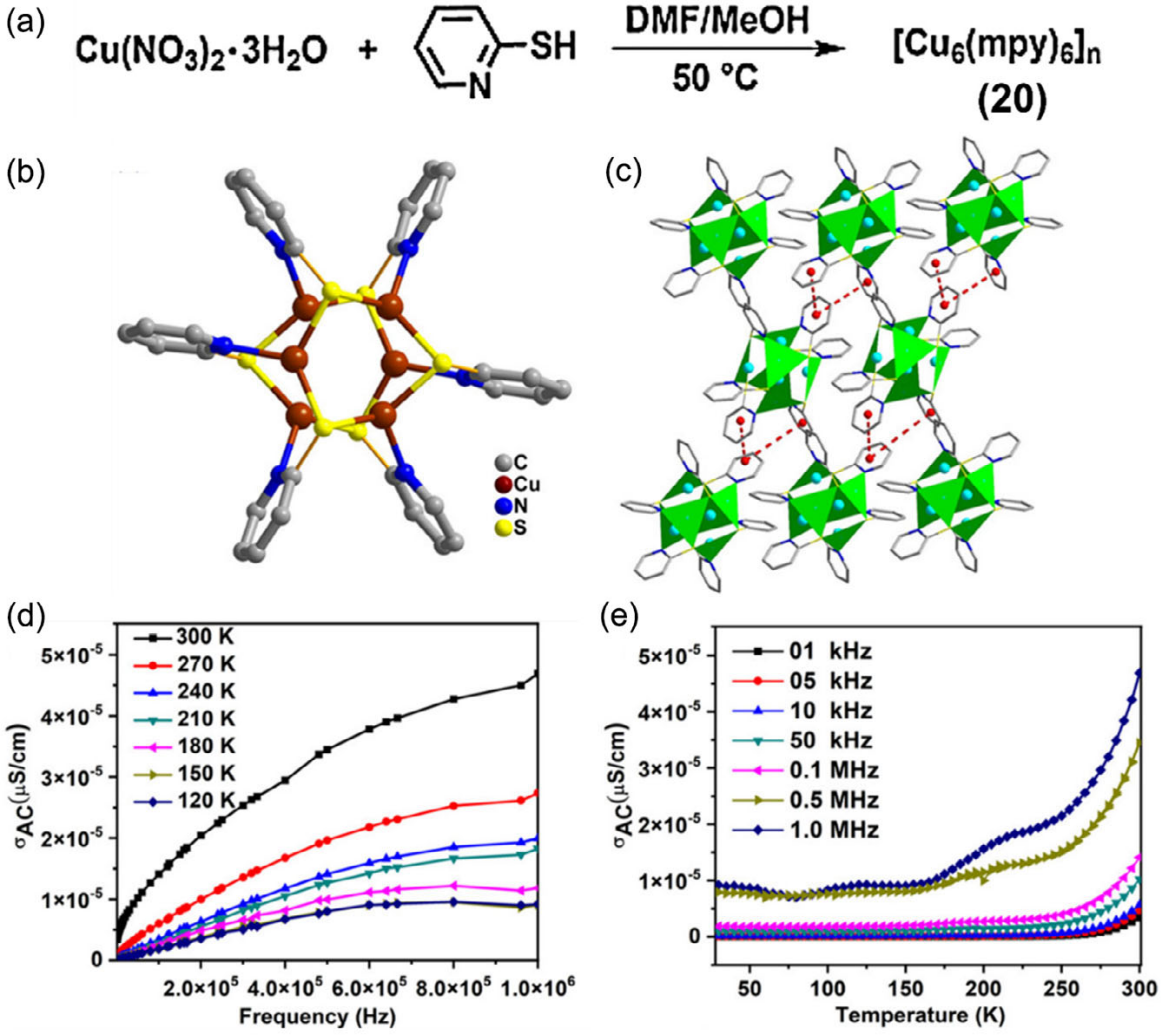
(a) Synthetic scheme of **20**. (b,c) Structural representation of **20**. (d) Frequency‐dependent AC conductivity of **20** at different temperatures. (e) Temperature‐dependent AC conductivity at different frequencies. Reproduced with permission from ref. [61]. Copyright 2022, American Chemical Society.

Emerging zero‐dimensional (0D) MOFs occupy an intermediate regime between molecular semiconductors and extended frameworks. A 0D copper‐based MOF, [Cu_6_(mpy)_6_]_*n*_ (**20**), where mpy **=** 2‐mercaptopyridine, featuring a paddle‐wheel‐like cluster architecture stabilized by strong Cu−S coordination, is recently reported (Figure 11a,b) [61]. Compound **20** displayed thermal stability up to 300°C, attributed to the strong coordination between the mercapto‐functionalized ligand and the Cu centers. The semiconductive behavior of MOF **20** was characterized by frequency‐ and temperature‐dependent conductivity (Figure 11d). AC conductivity increases from 3.38 × 10^–6^ to 4.69 × 10^–5^ S cm^–1^ between 1 kHz and 1 MHz, consistent with the presence of both free and trapped charge carriers. A progressive increase in temperature‐dependent conductivity was observed from 9.18 × 10^–6^ S cm^–1^ at 30 K to 4.69 × 10^–5^ S cm^–1^ at 300 K, confirming thermally activated semiconductive behavior. Together with the experimentally determined optical bandgap (1.9 eV), it is suggested that this behavior is likely facilitated by the favorable overlap between the p orbitals of the ligands and the d orbitals of the Cu centers, promoting charge delocalization through chemical bonding. Dielectric measurements reveal a high dielectric constant (κ ≈ 45) and a tanδ value of 1.38 at 1 kHz and 300 K, reflecting significant interfacial and ionic polarization. Although their conductivities remain modest (~10^–6^ S cm^–1^), their high dielectric constants and thermally activated transport suggest potential roles as dielectric–semiconductive hybrids, particularly in frequency‐dependent or memory‐related device concepts.

Taken together, these studies illustrate that MOFs can be deliberately positioned across the passive‐active spectrum of microelectronic materials, ranging from low‐κ insulating layers to semiconductive and conductive active components. Rather than representing parasitic leakage, electronic conductivity in MOFs can be precisely engineered through framework chemistry, dimensionality, and supramolecular interactions. Representative examples of semiconductive and highly cMOFs relevant to microelectronic integration are summarized in Table 3.

**TABLE 3:** Lists of semiconductive MOFs.

MOFs	Band gap, eV	Thermal stability, °C	Activation energy, *E*_a_, eV	Conductivity, σ, S/cm	Leakage current, A/mm^2^	Ref.
{[Ag_6_(S^*t*^Bu)_4_(TFBDC)]·guest}_*n*_	2.33	120	0.35	1.01 × 10^−8^ a2.29 × 10^−5^	n.r.	[62]
Cu_3_(btc)_2_⊃TCNQ	n.r.	n.r.	0.041	7 × 10^−2^	n.r.	[63]
[Zn_2_(TCPB)(BPDPNDI)]	n.r.	375	n.r.	5.8 × 10^−7^	n.r.	[64]
[Zn_2_(TCPB)(BPDPNDI)]⊃MV^2^	n.r.	n.r.	n.r.	2.3 × 10^−5^	n.r.	[64]
[Zn_2_(TCPB)(BPDPNDI)]⊃DNT	n.r.	n.r.	n.r.	1.5 × 10^−6^	n.r.	[64]
[Zn_2_(TCPB)(BPDPNDI)]⊃DFDNB	n.r.	375	n.r.	3.5 × 10^−6^	n.r.	[64]
[Zn_3_(lac)_2_(pybz)_2_]_*n*_⊃PPy	n.r.	400	n.r.	1.0 × 10^−2^	n.r.	[65]
NU‐901⊃C_60_	0.25	n.r.	n.r.	10^−3^	n.r	[66]
HoHHTP	0.765	400	0.25	0.05	n.r.	[67]
LaHHTP	0.85	400	0.26	0.82 × 10^−3^	n.r.	[67]
YbHHTP	0.73	400	0.25	0.01	n.r.	[67]
NdHHTP	0.82	400	0.24	0.8 × 10^−3^	n.r.	[67]
[K(TTFTC^+•^)H_2_]_*n*_	n.r.	200	0.22	1 × 10^–3^	n.r.	[68]
Mn_2_(TTFTB)	n.r.	425	n.r.	1.0 × 10^−4^	n.r.	[69]
Co_2_(TTFTB)	n.r.	400	n.r.	5.0 × 10^−5^	n.r.	[69]
Cd_2_(TTFTB)	n.r.	300	n.r.	6.79 × 10^−4^	n.r.	[69]
Zn_2_(TTFTB)	n.r.	n.r.	n.r.	2.03 × 10^−7^	n.r.	[69]
Cu[Cu(pdt)_2_]	n.r.	n.r.	n.r.	6.0 × 10^−4^	n.r.	[70]
I_2_@[Ag(TMT‐TTF)_0.5_NO_3_]_*n*_	n.r.	n.r.	0.08	4.5 × 10^−3^	n.r.	[71]
Cu(TCNQ)	n.r.	n.r.	n.r.	10^−1^	n.r.	[72]
Cu_3_(BHT) (under He)	n.r.	n.r.	0.00206	1580	n.r.	[73]
Cu_3_(BHT)	n.r.	n.r.	n.r.	2500	n.r.	[74]
[Ag_5_(BHT)]_*n*_	n.r	n.r.	0.16	250	n.r.	[75]
Fe‐PTC	0.2	300	0.2	10	n.r.	[76]
[Cu_2_(Hmna)(mn)][NH_4_]	1.34	340	0.006	10.96	n.r.	[4]
Cu_3_(HHTP)_2_	n.r.	n.r.	n.r.	0.02	n.r.	[77]
Cu_3_(BHSe)	n.r.	420	n.r.	110	n.r.	[78]
Ni_2_[Ni(Pc)]	n.r.	n.r.	0.0011	0.2	n.r.	[79]
[Sr(ntca)(H_2_O)_2_]·H_2_O}_*n*_	n.r.	n.r.	n.r.	1 × 10^−4^	n.r.	[1]
[Sr(H^124^btc)(H_2_O)]_*n*_	2.3	520	0.17	6.5 × 10^−6^	n.r.	[54]
[Sm_2_(bhc)(H_2_O)_6_]_*n*_	n.r.	455	n.r.	1.46 × 10^−5^	8.13 × 10^−12^	[37]
[Sr_2_(1,3‐bdc)_2_(H_2_O)(DMF)]_*n*_	n.r.	550	n.r.	10^−7^	n.r.	[39]
[Cu_6_O_4_(H_2_O)_4_(HFDP)_2_]·4H_2_O	n.r.	152	n.r.	1.899 × 10^−7^	n.r.	[3]
[Cu_6_O_4_(H_2_O)_4_(HFDP)_2_]·4H_2_O (guest free)	n.r.	188	n.r.	2.624 × 10^−7^	n.r.	[3]
[Cu_6_O_4_(H_2_O)_4_(HFDP)_2_]·4H_2_O (guest and coordinated H_2_O free)	n.r.	267	n.r.	2.431 × 10^−7^	6.42 × 10^–12^	[3]

Abbreviations: 1,3‐bdc = benzene‐1,3‐dicarboxylate; H4TTFTB, tetrathiafulvalene tetrabenzoate; bhc = benzenehexacarboxylate; BHSe = benzenehexaselenolate; BHT, benzenehexathiol; BPDPNDI = N, N′‐bis(4‐pyridyl)−2,6‐dipyrrolidyl naphthalenediimide; btc = benzene‐1,3,5‐tricarboxylate; H_6_HHT*P* =  2,3,6,7,10,11‐hexahydroxytriphenylene; H_3_^124^btc = 1,2,4‐benzenetricarboxylic acid; HFD*P* = 4,4′‐(hexafluoroisopropylidene)diphthalate; HHT*P* = 2,3,6,7,10,11‐hexahydroxytriphenylene; Hmna = 6‐mercaptonicotinic acid, mn = 6‐mercaptonicotinate; lac = D, L, lactate; n.r = not reported; ntca = 1,4,5,8‐naphthalenetetracarboxylate; PC = 2,3,9,10,16,17,23,24‐*octa*amino‐phthalocyaninate; pdt = 2,3‐pyrazinedithiolate; ppy = polypyrrole; PTC = 1,2,3,4,5,6,7,8,9,10,11,12‐perthiolated coronene; pybz = 4‐pyridylbenzoate; TCNQ = 7,7,8,8‐tetracyanoquinododimethane; TCPB = 1,2,4,5‐tetrakis(4‐carboxyphenyl)benzene; TFBDC = 0.2,3,5,6‐tetrafluorobenzene‐1,4‐dicarboxylate; TMTTTF, tetrakis(methylthio) tetrathiafulvalene; TTF‐TC, tetrathiafulvalenetetracarboxylate.

a 100°C.

### 2.3 White‐Light Emission Property of MOFs

The development of environmentally benign, efficient, and structurally robust single‐component white‐light‐emitting MOFs has emerged as a compelling strategy for next‐generation WLED technologies. Unlike multicomponent phosphor‐converted or dopant‐based systems, single‐component MOFs inherently circumvent challenges associated with energy transfer inefficiency, fluorescence self‐absorption, spectral instability, and batch‐to‐batch variability. From a materials chemistry perspective, the integration of broadband emission within a single crystalline framework offers simplified device architectures, improved photophysical reproducibility, and enhanced thermal and mechanical stability.

**FIGURE 12:**
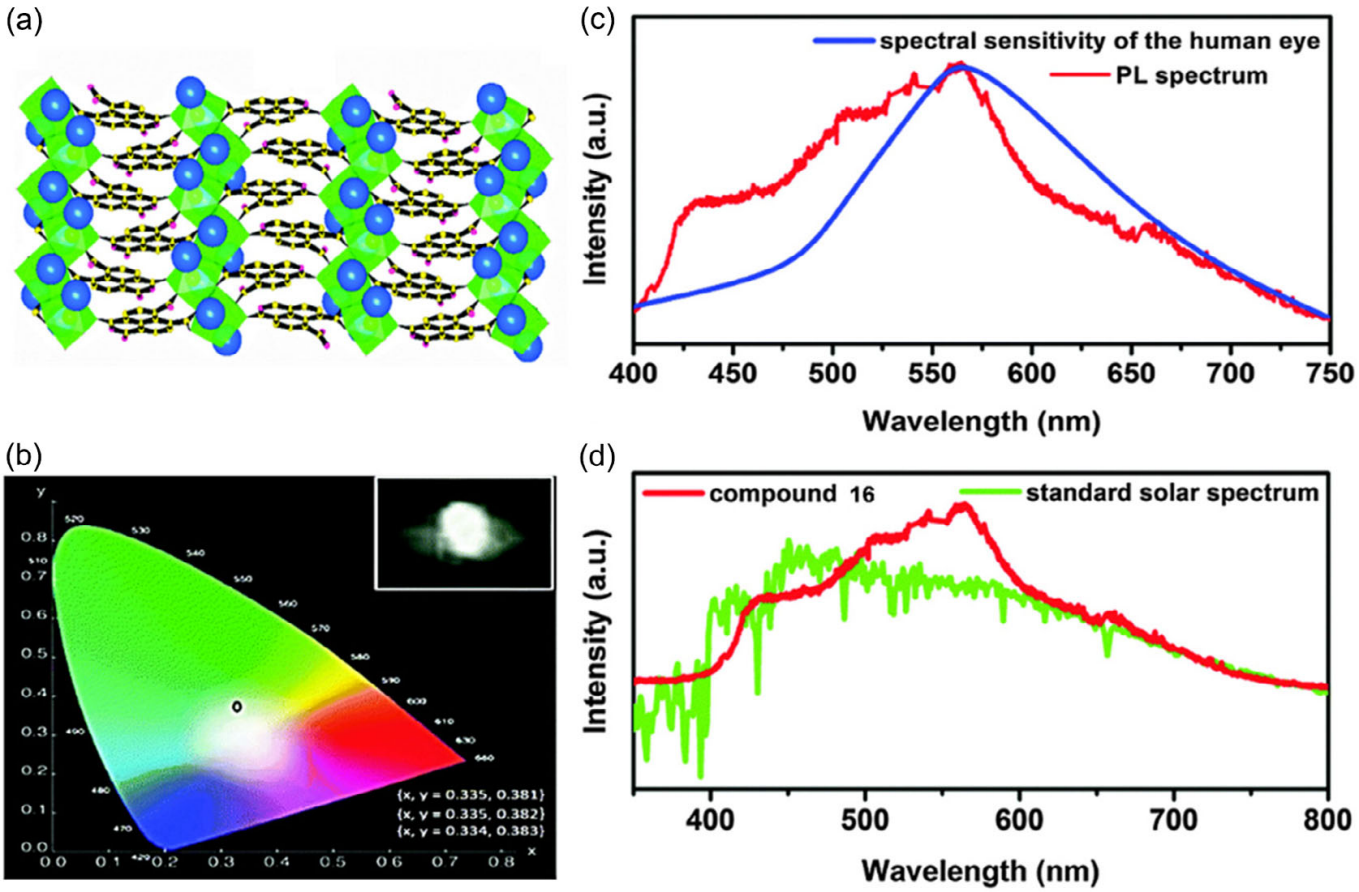
(a) Structural representation of **16**. (b) CIE color coordinates correspond to various laser powers (27 to 29 μW), a real‐time photograph of the sample during PL measurements. (c) Comparison between the photoluminescence (PL) spectrum of **16** and the spectral sensitivity of a human eye during the daytime. (d) Emission range of **16** compared with that of the standard solar light. Reproduced with permission from ref. [80]. Copyright 2016, Royal Society of Chemistry.

Among the reported systems, alkaline earth metal‐based MOFs have attracted particular attention due to their low toxicity, earth abundance, and favorable coordination environments that promote ligand‐centered and charge‐transfer emissions without reliance on rare‐earth dopants. In 2016, the first electrically driven, continuous white‐light‐emitting device based on a strontium MOF, {[Sr(ntc)(H_2_O)_2_]·H_2_O}_*n*_ (**16**, ntc = 1,4,5,8‐naphthalenetetracarboxylate) was reported [80]. This framework crystallizes with Sr(II) centers in a dodecahedral coordination environment, bound to ntc^2−^ ligands and coordinated water molecules (Figure 12a). The framework is stabilized by an extensive hydrogen‐bonding network, supplemented by π–π stacking and CH–π interactions, which together suppress nonradiative decay pathways.

Under pulsed 266 nm excitation, compound **16** exhibits a broad and continuous photoluminescence (PL) spectrum spanning 400–750 nm, with a maximum at ~550 nm. The emission originates from synergistic contributions of ligand‐centered transitions, metal‐centered states, and MLCT/LMCT processes. The resulting white emission closely resembles natural sunlight, yielding CIE chromaticity coordinates of (0.335, 0.381)−(0.334, 0.383) and CCT values of ~5400−5450 K over a narrow excitation power window (27−29 μW) (Figure 12b–d). The measured quantum efficiency (QE) of ~10% is notable for a single‐component MOF emitter and highlights the effectiveness of cooperative emission pathways within the framework.

Building upon these photophysical characteristics, electrically driven white‐light electroluminescence (EL) by integrating MOF **16** with graphene to optimize interfacial band alignment was demonstrated [1]. The EL device architecture comprised a ZnO electron‐transport layer deposited on a Si/SiO_2_/Ag substrate, coated with a thin film of MOF **16**, and capped with a transparent graphene/PMMA electrode. The use of single‐layer graphene simultaneously enabled efficient charge injection and mitigated short‐circuiting issues common in metal‐electrode configurations. Upon electrical excitation, the EL spectrum exhibited multiple emission bands at 410, 506.5, 560, and 644 nm, corresponding to interligand, intermetallic, and charge‐transfer transitions, in close agreement with PL measurements.

**FIGURE 13:**
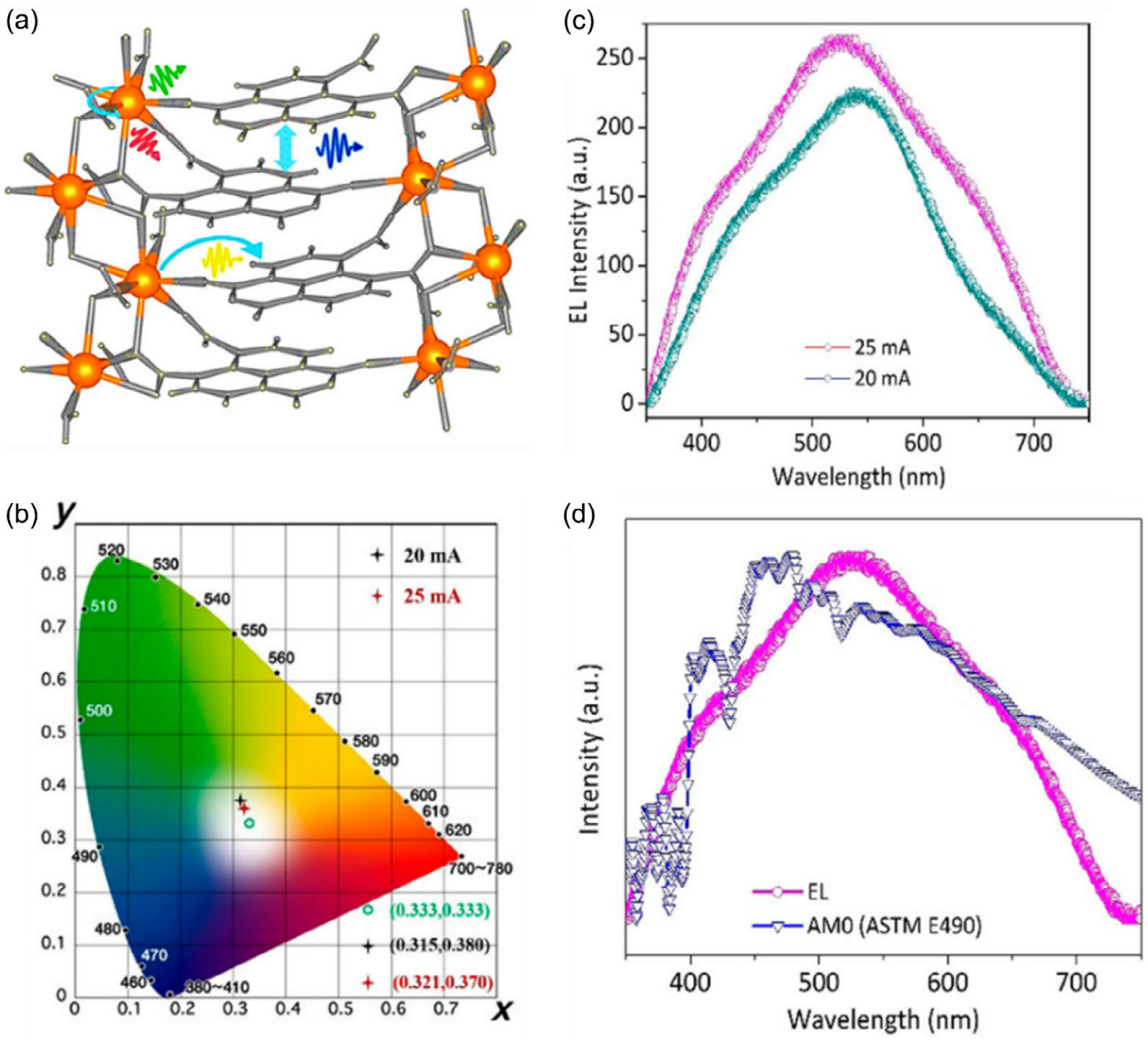
(a) Schematic illustration of different colors of emissions from **16**. (b) CIE chromaticity diagram of the EL emissions. The circle corresponds to the coordinates (0.333, 0.333). (c) EL spectra of the device at injection currents of 20 and 25 mA. (d) Comparison of the EL spectrum with natural sunlight. Reproduced with permission from ref. [1]. Copyright 2016, American Chemical Society.

Thermal annealing at 250°C selectively suppressed the MLCT contribution, indicating that coordinated and guest water molecules play a decisive role in enabling charge‐transfer‐mediated emission. Time‐resolved PL measurements revealed both single‐ and dual‐exponential decay components, confirming the coexistence of multiple radiative pathways (Figure 13a). The resulting EL output produced visually comfortable white light (*λ*_EL_^max^ = 555 nm) with CIE coordinates of (0.327, 0.367), a CCT of ~5400 K, and a QE of 1.2%. Importantly, the emission spectrum exhibited significantly reduced blue‐light intensity relative to commercial WLEDs, offering potential advantages in ocular safety. This work represents one of the rare demonstrations of direct white‐light EL from a single‐component solid‐state emitter (Figure 13c,d).

Time‐resolved PL measurements were performed for **16** at the emission maxima to confirm the origin of the PL bands. The peak at 435 nm shows a short lifetime of ~0.60 ± 0.20 ns, closely matching the free H_4_ntc ligand lifetime of ~0.98 ± 0.20 ns, confirming ligand‐centered fluorescence. The emissions at 507, 568, and 640 nm exhibit dual lifetimes, where the shorter components arise from metal‐based transitions and the longer ones from MLCT, all remaining in the nanosecond range. These nanosecond‐scale lifetimes confirm that the observed PL originates from fluorescence rather than phosphorescence.

The electrochemical and operational stability of MOF **16** were also evaluated. The electrochemical stability was confirmed by consistent *I*–*V* curves measured on a single‐crystalline sample with Au electrodes (~200 μm gap) before and after applying a constant 15 V bias for 6 h under ambient conditions. Furthermore, the device maintained stable luminescence efficiency with negligible degradation for more than 2 months in ambient environment. This excellent stability is attributed to the inherent robustness of the MOF and the effective passivation provided by the top graphene/PMMA layer, whose dense structure with small lattice constant prevents penetration of external molecules into the porous MOF framework.

Beyond lighting applications, hybrid films of MOF **16** and graphene deposited on flexible PDMS substrates exhibited high carrier mobility and broadband photodetection capability spanning the UV–visible region [81]. Furthermore, the strong optical feedback arising from the framework's random microstructure enabled lasing at ultra‐low thresholds, attributed to multiple scattering and light‐trapping effects [82], an observation that further underscores the multifunctional optoelectronic potential of this class of materials.

**FIGURE 14:**
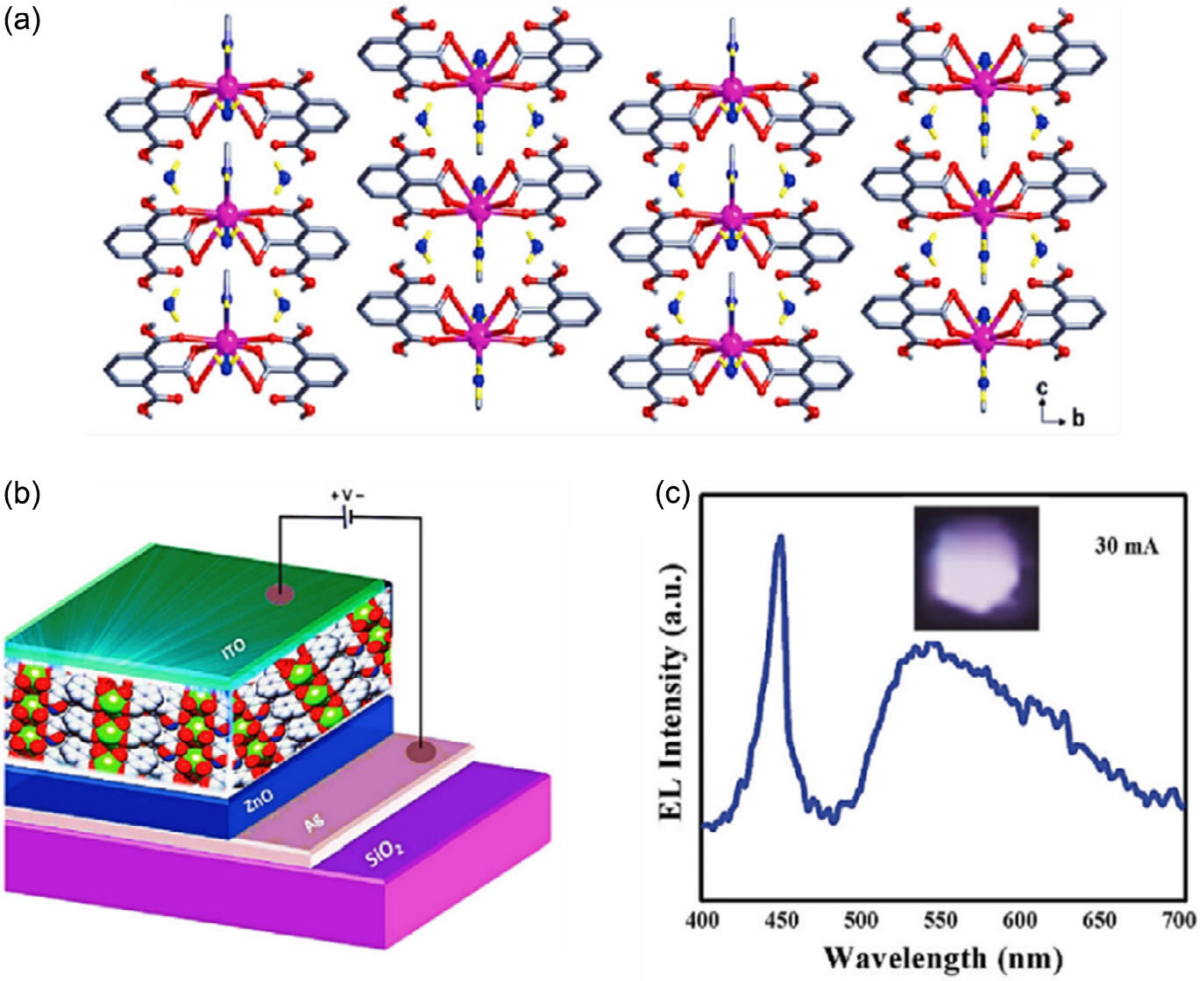
(a) The structural representation of **17** shows each unit connected in an ABAB fashion, which is extended into 2D sheets. (b) Schematic illustration of a visible broadband LED device where the *p*–*n* junction is formed by ZnO (155.5 nm) and Sr‐MOF on the top of 199.6 nm Ag film. The p‐type Si/SiO_2_ and ITO were used as the substrate and the top electrode, respectively (inset shows an optical photo of the light emissions from the device taken using a mobile camera). (c) EL spectrum of the Sr MOF‐based LED device. Reproduced with permission from ref. [57]. Copyright 2019, American Chemical Society.

Polycarboxylate ligands offer additional opportunities for tailoring emission through structural reorganization and intermolecular interactions. A butterfly‐like coordination polymer {[Sr(H_2_^123^btc)_2_(MeOH)(H_2_O)_2_]·2H_2_O} (**17**) was synthesized using benzene‐1,2,3‐tricarboxylic acid under hydrothermal conditions (Figure 14a) [57]. Single‐crystal X‐ray diffraction revealed a distorted trigonal coordination environment around Sr(II), leading to a 2D ABAB‐stacked architecture stabilized by hydrogen bonding. Under 266 nm excitation, compound **17** exhibits emission peaks at 440, 480, and 510 nm, assigned to LMCT, metal‐centered, and ligand‐centered transitions, yielding CIE coordinates of (0.19, 0.25). Compared with the lifetime (< 1 ns) of the free ligand emission, the peak at 440 nm displays a longer lifetime of ~2 ns, attributed to intersystem charge‐transfer from ligand to metal. The peaks at 480 and 510 nm show short lifetime (< 1 ns), corresponding to metal‐ and ligand‐center emissions, respectively. These nanosecond‐scale lifetimes are consistent with reported values for ligand‐, metal‐, and charge‐transfer emissions in MOFs, supporting the deconvoluted PL spectra.

Removal of lattice solvents induces a bandgap reduction from 2.52 to 2.0 eV, accompanied by changes in emission intensity and spectral distribution, consistent with framework reorganization. These observations are supported by DFT calculations, which reveal solvent‐dependent modulation of the electronic structure. A single‐layer EL device incorporating compound **17** between an ITO electrode and a ZnO/Si/SiO_2_/Ag substrate exhibited cool white emission composed of violet (448 nm) and yellow (544 nm) components (Figure 14b). The optoelectronic performance of the device showed a QE of approximately 1.0%, whereas the intrinsic PL QE of the compound was significantly higher at around 14%. The emitted light showed a CCT of 5620 K, CIE coordinates of (0.33, 0.32), along with a high CRI of 96. These results position compound **17** among the most efficient single‐component MOF‐based white emitters reported to date.

The material exhibited excellent electrochemical stability, as evidenced by a nearly unchanged *I*–*V* curve after continuous 20 V bias for 8 h under ambient conditions. Furthermore, the device maintained consistent luminescence efficiency with negligible degradation for more than 70 days in ambient environment. This high stability is attributed to the inherent electrochemical and optical robustness of the compound, the superior ZnO film quality formed by RF‐sputtering, and protection by the top ITO/glass layer.

Expanding this design paradigm, a new class of phosphor‐free alkaline‐earth MOF emitters has been developed to further enhance performance and durability. The barium‐based MOF [Ba(2,6‐ndc)(H_2_O)_2_]·H_2_O (**21**, 2,6‐ndc = 2,6‐naphthalenedicarboxylate) was synthesized hydrothermally at 140°C and exhibits high thermal stability up to 490°C [83]. Structural analysis revealed trigonal‐prismatic Ba(II) coordination, reinforced by strong π–π stacking interactions (3.23–3.77 Å) and an extensive hydrogen‐bonding network.

**FIGURE 15:**
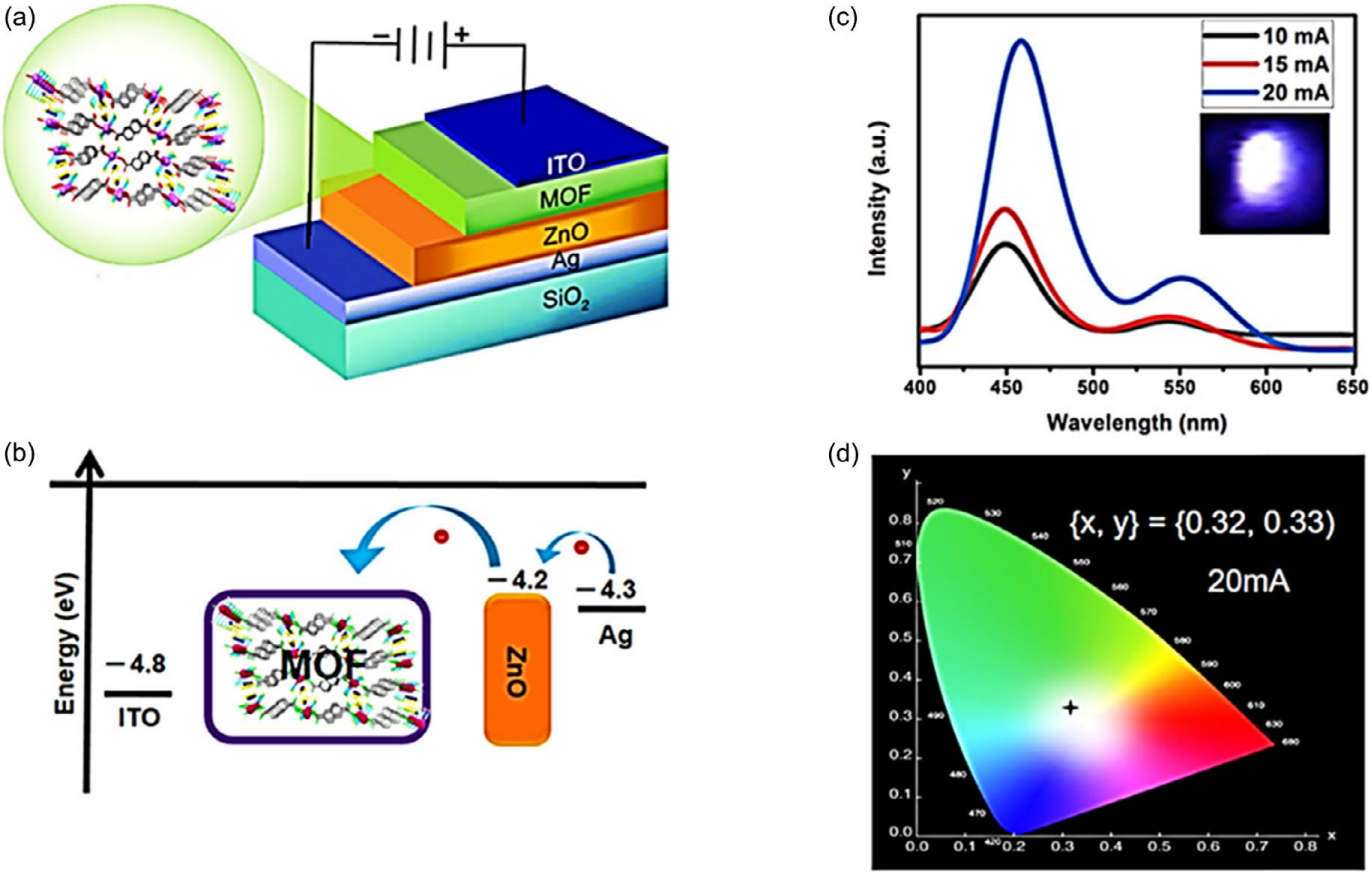
(a) Schematic representation of the developed LED device. (b) Energy band diagram for **21**. (c) EL spectrum of the Ba‐MOF‐based device under different forward injection currents, as shown in the inset. (d) CIE chromaticity coordinates of **21** *.* Reproduced with permission from ref. [83]. Copyright 2021, American Chemical Society.

Under 374 nm excitation, compound **21** displays intense broadband PL spanning 400–700 nm, with a remarkably high QE of 75%. The emission arises from combined ligand‐centered, metal‐centered, and charge‐transfer transitions, as corroborated by time‐resolved spectroscopy and DFT calculations. EL devices fabricated using nanometer‐scale films of MOF **21** on ZnO exhibited white emission peaks at 450 and 550 nm, yielding CIE coordinates of (0.32, 0.33), a CCT of 6113 K, a CRI of 84, and a QE of 3.06% (Figure 15).

The long‐term stability of the Ba‐MOF device was evaluated by monitoring its EL efficiency at different time intervals. The device performance remained essentially unchanged in ambient conditions for more than 80 days. This high stability is attributed to the optical, thermal, and electrochemical robustness of MOF **21** as well as the excellent ZnO film quality obtained by RF‐sputtering instead of spin‐coating.

Collectively, these results demonstrate that alkaline earth metal‐based MOFs can serve as efficient, stable, and environmentally sustainable single‐component white‐light emitters, capable of both photoluminescent and electroluminescent operation. The ability to engineer broadband emission through framework design, intermolecular interactions, and controlled charge‐transfer processes positions these materials as strong candidates for next‐generation solid‐state lighting.

Based on the above discussion, it is evident that phosphor‐free MOF‐based white‐light emitters represent a promising alternative approach for solid‐state lighting. These devices generate broadband white emission directly from the hybrid inorganic–organic framework through a combination of ligand‐centered π–π transitions and MLCT or LMCT processes, without requiring any secondary conversion layer. This intrinsic emission strategy eliminates energy losses associated with Stokes shifts, potentially enhances long‐term photostability due to the rigid crystalline architecture of MOFs, and offers exceptional structural tunability through the rational design of metal nodes, organic linkers, and guest molecules. Such molecular‐level spectral tunability enables precise optimization of key performance metrics, including CIE chromaticity coordinates, CRI, CCT, and photoluminescence quantum yield (PLQY). A single MOF can, in principle, replace a multi‐phosphor blend and reduce color drift caused by different red/green/blue phosphors aging at different rates. Currently some examples have already shown promising potential in this regard albeit to date state‐of‐the‐art phosphor‐free MOF emitters typically exhibit lower external QE compared to established commercial white LEDs and, in some cases, still require improvement in overall color quality. A pressure‐treated Zn‐IPA MOF was reported with white‐light PLQY of 81.3% and CIE coordinates of (0.29, 0.37) [84]. Another MOF phosphor, CdCl_2_(acridine), showed 65% quantum yield, retained 95% emission after 60 days in water, and retained 84% intensity after heating to 150°C [85].

As a phosphor‐free emitter, MOFs must act as the active electroluminescent layer while most of them have poor charge mobility. This creates several efficiency penalties including high operating voltage, Joule heating, poor carrier injection, exciton‐polaron quenching, and low external QE. They can be improved by enhancing electrical conductivity of phosphor‐free MOFs.

Unlike commercial solid‐state phosphors such as YAG:Ce, LuAG:Ce, nitride phosphors, and ceramic phosphor plates that can survive harsh LED or laser‐lighting conditions, MOFs are possible to undergo degradation, oxidation, photochemical changes under intense blue/UV pumping. In addition, moisture and thermal stability of MOFs is highly framework‐dependent. Hydrolysis of metal‐ligand bonds, guest leaching, pore collapse, and ligand exchange can reduce emission. For comparison, inorganic oxide and many nitride phosphors are of excellent moisture and thermal stability, and long‐term durability, while KSF/PFS:Mn^4+^ fluorides need moisture protection.

These limitations highlight the need for further advancements in charge carrier mobility, efficient exciton harvesting and management, interfacial engineering, and device architecture to fully realize the potential of MOF‐based white‐light sources.

A comparative summary of photophysical parameters and device metrics for representative MOF‐based white emitters is provided in Table 4.

**TABLE 4:** Representative examples of white‐light emitting MOFs and derived composites.

MOF or MOF⊃Guest	Excitation, nm	Quantum yield, %	CIE	CCT, K	CRI	Ref.
{[Zn(5‐ipa)(Py)_2_]·2H_2_O}∞	260	32.5	(0.31, 0.33)	n.r.	n.r.	[86]
[Sr(H_2_^123^btc)_2_(MeOH)(H_2_O)_2_]·2H_2_O	266	14	(0.33, 0.32)	5620	96	[57]
[Ag(cpa)]_*n*_·nH_2_O	349	10.86	(0.33, 0.34)	n.r.	n.r.	[87]
TMOF‐5(Cl)	380	6–8	(0.36, 0.40)	4784	85	[88]
ZIF‐8	300	4.73	(0.326, 0.390)	5754	n.r.	[89]
	320		(0.319, 0.371)	6045		
	340		(0.301, 0.346)	7002		
	365		(0.290, 0.339)	7697		
	380		(0.289, 0.345)	7667		
	400		(0.290, 0.361)	7411		
TMOF‐5(Br)	380	1.5	(0.37, 0.39)	4258	89	[88]
{[H_2_N(CH_3_)_2_]_6_[Zn_16_(btc)_12_(gac) (DMA)_3_(H_2_O)_3_]}·17DMA	300	1.36	(0.320, 0.373)	5736 to 6020^b^	97	[90]
	330		(0.318, 0.363)			
	360		(0.298, 0.333)			
	390		(0.297, 0.346)			
{[Sr(ntca)(H_2_O)_2_]·H_2_O}_*n*_	266	1.2	(0.327, 0.367)	5400	n.r.	[1]
[Pb_2_(pia)_2_(DMA)]·DMA	350	0.92	(0.332, 0.347)	5696	95	[91]
TMOF‐5(I)	380	n.r.	(0.40, 0.48)	3972	70	[88]
ZJU‐28⊃C6/R6G/R101	460	82.9	(0.36, 0.34)	4446	88	[92]
HSB‐W1⊃R‐phycoerythrin (R‐PE)	405	60.0	(0.33, 0.34)	5740	85	[93]
[Zn_4_OL_2_·*x*DMF]_*n*_⊃DCM/C6	365	39.4	(0.32, 0.31)	6186	91	[94]
Gd‐cba MOF⊃RB	320	39.2	(0.34, 0.33)	5152	n.r.	[95]
Zr‐MOF⊃carbon dots (CDs)	365	37	(0.31, 0.34)	n.r.	82	[96]
[(CH_3_)_2_NH_2_]_15_[(Cd_2_Cl)_3_(TATPT)_4_] ·12DMF·18H_2_O⊃[In(ppy)_2_(bpy)]^+^	370	20.4	(0.31, 0.33)	5900	80	[97]
{Cd(PyTa)(cba)_2_(MeOH)(H_2_O)}∞ (CP1)⊃CdTe QDs	330	18.0	(0.33, 0.32)	5622	n.r.	[98]
ZJU‐28⊃DSM/AF	365	17.4	(0.34, 0.32)	5327	91	[99]
NENU‐521⊃Alq_3_	370	11.4	(0.291, 0.327)	7796	n.r.	[100]
[(Eu_1.22_Tb_0.78_(1,4‐phda)_3_(H_2_O)] (H_2_O)_2_⊃carbon dots (CDs)	370	9.0	(0.334, 0.334)	5443	93	[101]
In(BTB)_2/3_(OA)(DEF)_3/2_(SMOF‐1)⊃10%Eu^3+^	350	4.3^a^	(0.369, 0.301)	3606	63	[102]
	360		(0.285, 0.309)	7068	81	
	380		(0.304, 0.343)	8695	93	
	394		(0.309, 0.298)	6839	93	
[La(H_2_O)_4_(pdc)]_4_}[SiMo_12_O_40_]·2H_2_O⊃[La_0.95_Dy_0.045_Eu_0.005_(H_2_O)_4_(pdc)]_4_} [SiMo_12_O_40_]·2H_2_O	295	n.r.	(0.361, 0.3408)	n.r.	n.r.	[103]
[La(H_2_O)_4_(pdc)]_4_}[SiMo_12_O_40_]·2H_2_O⊃{La_0.95_Eu_0.05_(H_2_O)_4_(pdc)]_4_} [SiMo_12_O_40_]·2H_2_O	295	n.r.	(0.3425, 0.2458)	n.r.	n.r.	[103]
[La(H_2_O)_4_(pdc)]_4_}[SiMo_12_O_40_]·2H_2_O⊃La_0.95_Eu_0.04_Tb_0.01_(H_2_O)_4_(pdc)]_4_} [SiMo_12_O_40_]·2H_2_O	295	n.r.	(0.3857, 0.3377)	n.r.	n.r.	[103]
[Eu(MCTCA)_1.5_(H_2_O)_2_]·1·75H_2_O ⊃H_4_TBAPy	350	n.r.	(0.3482, 0.3301)	n.r.	n.r.	[104]
ZIF‐8⊃C‐151 + ZIF‐8⊃F + ZIF‐8⊃RB	365	n.r.	(0.32, 0.34)	n.r.	n.r.	[105]
ZJU‐28⊃DSM/AcrM	365	n.r.	(0.34, 0.32)	5004	86	[99]
[Me_2_NH_2_][In(bptc)]·solvents⊃Safranin O	380	n.r.	(0.32, 0.33)	n.r.	n.r.	[106]

Abbreviations: 5‐ipa = 5‐azidoisopthalate; C6 = coumarin 6; R6G, Rhodamine 6G; ^123^btc = benzene‐1,2,3‐tricarboxylate; AcrM = 10‐methyl‐acridine; AF, acriflavine; AF, acriflavine; bptc = biphenyl‐3,3′, 5,5′‐tetracarboxylate; bpy = 2,2′‐bipyridine; BTB = 1,3,5‐tris(4‐carboxyphenyl)benzene; C‐151 = 7‐amino‐4‐(trifluoromethyl)coumarin; cba = 4‐(2‐carboxyvinyl benzoate); CCT, correlated color temperature; CRI, color rendering index; DCM = 4‐(dicyanomethylene)‐2‐methyl‐6‐(*p*‐dimethylaminostyryl)‐4H, pyran; DEF = N, N′‐diethylformamide; DMA = N, N’‐dimethylacetamide; DSM = 4‐(*p*‐dimethylaminostyryl)‐1‐methylpyridinium; DSM = 4‐(*p*‐dimethylaminostyryl)‐1‐methylpyridinium; F, fluorescein; H_6_TATPT = 2,4,6‐tris(2,5‐dicarboxylphenylamino)−1,3,5‐triazine; H_4_TBAPy = 1,3,6,8‐tetrakis (*p*‐benzoic acid)pyrene; H_3_btc = 1,3,5‐benzenetricarboxylic acid; H_2_gac = glycolic acid; HSB, hydrogenated Schiff base; L = 4,4′, 4′’‐(9H, carbazole‐3,6,9‐triyl)‐tribenzoate; MCTCA = 5‐methyl‐1‐(4‐carboxylphenyl)‐1H‐1,2,3‐triazole‐4‐carboxylate; ntca = 1,4,5,8‐naphthalenetetracarboxylate; OA, oxalic acid; phda = 1,4‐phenylenediacetate; pia = 5‐(pyridin‐4‐yl)isophthalate; ppy = 2‐phenylpyridine; Py = pyridine; PyTa = 2,3‐dihydroxy‐N1, N4‐di(pyridine‐3‐yl)‐succinimide; R101 = Rhodamine 101; RB, rhodamine B.

a *λ*_ex_ = 330 nm.

b different excitations at 300–420 nm.

## 3 Conclusions and Perspectives

This review establishes MOFs as a rapidly emerging class of functional materials for microelectronic and optoelectronic applications. Progress in molecular design, bandgap engineering, and synthetic control has enabled MOFs to operate as low‐ and high‐κ dielectrics, semiconductors with enhanced charge transport, and intrinsic broadband white‐light emitters. Precise modulation of framework composition, through metal‐node selection, π‐conjugated linkers, guest incorporation, and structural water, has proven central to tuning electronic structure and photophysical response. Notably, recent advances in electrically driven, phosphor‐free white‐light‐emitting MOFs, particularly single‐component systems, highlight a viable route toward simplified device architectures and environmentally sustainable luminescent technologies.

Despite these achievements, further improvements in electrical conductivity, operational stability, and scalable device integration remain critical. Future efforts should prioritize framework dimensionality control, reinforcement of through‐bond and through‐space charge transport pathways, and the systematic use of predictive computational methods, including DFT, to guide structure‐property optimization.

Taken together, the inherent tunability, multifunctionality, and processing versatility of MOFs position them as compelling candidates for next‐generation nanoelectronic and optoelectronic systems, with the potential to enable miniaturized, energy‐efficient, and sustainable device platforms beyond the limits of conventional materials.

## Funding

This study was supported by Ministry of Science and Technology, Taiwan (106‐2113‐M‐001‐032, 109‐2811‐M‐030‐500, 109‐2113‐M‐030‐011, 110‐2113‐M‐030‐011) and Natioal Science and Technology Council, Taiwan (113‐2113‐M‐030‐002).

## Conflicts of Interest

The authors declare no conflicts of interest.

## Data Availability

The data that support the findings of this study are available from the corresponding author upon reasonable request.

## References

[1] G. Haider , M. Usman , T.‐P. Chen , P. Perumal , K.‐L. Lu , and Y.‐F. Chen , “Electrically Driven White Light Emission from Intrinsic Metal–Organic Framework,” ACS Nano 10 (2016): 8366–8375. 27576847 10.1021/acsnano.6b03030

[2] M. Usman , C. H. Lee , D. S. Hung , et al., “Intrinsic Low Dielectric Behaviour of a Highly Thermally Stable Sr‐Based Metal–organic Framework for Interlayer Dielectric Materials,” Journal of Materials Chemistry C 2 (2014): 3762–3768.

[3] A. I. Inamdar , A. Pathak , M. Usman , et al., “Highly Hydrophobic Metal–organic Framework for Self‐Protecting Gate Dielectrics,” Journal of Materials Chemistry A 8 (2020): 11958–11965.

[4] A. Pathak , J.‐W. Shen , M. Usman , et al., “Integration of a (–Cu–S–)*n* Plane in a Metal–organic Framework Affords High Electrical Conductivity,” Nature Communications 10 (2019): 1721. 10.1038/s41467-019-09682-0 PMC6461620 30979944

[5] N. Stock and S. Biswas , “Synthesis of Metal‐Organic Frameworks (MOFs): Routes to Various MOF Topologies, Morphologies, and Composites,” Chemical Reviews 112 (2012): 933–969. 22098087 10.1021/cr200304e

[6] A. J. Howarth , Y. Liu , P. Li , et al., “Chemical, Thermal and Mechanical Stabilities of Metal–organic Frameworks,” Nature Reviews Materials 1 (2016): 15018.

[7] S. Yuan , L. Feng , K. Wang , et al., “Stable Metal–Organic Frameworks: Design, Synthesis, and Applications,” Advanced Materials 30 (2018): 1704303. 10.1002/adma.201704303 29430732

[8] R. Farrell , T. Goshal , U. Cvelbar , N. Petkov , and M. A. Morris , “Advances in Ultra Low Dielectric Constant Ordered Porous Materials,” The Electrochemical Society Interface 20 (2011): 39–46.

[9] M. Usman , S. Mendiratta , and K.‐L. Lu , “Semiconductor Metal–Organic Frameworks: Future Low‐Bandgap Materials,” Advanced Materials 29 (2017): 1605071. 10.1002/adma.201605071 27859732

[10] P. Thanasekaran , C.‐H. Su , Y.‐H. Liu , and K.‐L. Lu , “Weak Interactions in Conducting Metal–organic Frameworks,” Coordination Chemistry Reviews 442 (2021): 213987.

[11] E. M. Johnson , S. Ilic , and A. J. Morris , “Design Strategies for Enhanced Conductivity in Metal–Organic Frameworks,” ACS Central Science 7 (2021): 445–453. 33791427 10.1021/acscentsci.1c00047 PMC8006162

[12] C. Li , L. Zhang , J. Chen , et al., “Recent Development and Applications of Electrical Conductive MOFs,” Nanoscale 13 (2021): 485–509. 33404574 10.1039/d0nr06396g

[13] H. Meng , Y. Han , C. Zhou , et al., “Conductive Metal–Organic Frameworks: Design, Synthesis, and Applications,” Small Methods 4 (2020): 2000396.

[14] L. S. Xie , G. Skorupskii , and M. Dincă , “Electrically Conductive Metal–Organic Frameworks,” Chemical Reviews 120 (2020): 8536–8580. 32275412 10.1021/acs.chemrev.9b00766 PMC7453401

[15] X. Xiao , L. Zou , H. Pang , and Q. Xu , “Synthesis of Micro/Nanoscaled Metal–organic Frameworks and Their Direct Electrochemical Applications,” Chemical Society Reviews 49 (2020): 301–331. 31832631 10.1039/c7cs00614d

[16] L.‐H. Xie , M.‐M. Xu , X.‐M. Liu , M.‐J. Zhao , and J.‐R. Li , “Hydrophobic Metal–Organic Frameworks: Assessment, Construction, and Diverse Applications,” Advanced Science 7 (2020): 1901758. 32099755 10.1002/advs.201901758 PMC7029650

[17] L. Guo , J. Sun , J. Wei , Y. Liu , L. Hou , and C. Yuan , “Conductive Metal‐Organic Frameworks: Recent Advances in Electrochemical Energy‐Related Applications and Perspectives,” Carbon Energy 1 (2020): 1–20.

[18] M. Ding , X. Cai , and H.‐L. Jiang , “Improving MOF Stability: Approaches and Applications,” Chemical Science 10 (2019): 10209–10230. 32206247 10.1039/c9sc03916c PMC7069376

[19] Y. Tang , H. Wu , W. Cao , Y. Cui , and G. Qian , “Luminescent Metal–Organic Frameworks for White LEDs,” Advanced Optical Materials 9 (2021): 2001817.

[20] P. Li , Z. Zhou , Y. S. Zhao , and Y. Yan , “Recent Advances in Luminescent Metal–organic Frameworks and Their Photonic Applications,” Chemical Communications 57 (2021): 13678–13691. 34870655 10.1039/d1cc05541k

[21] W. P. Lustig and J. Li , “Luminescent Metal–organic Frameworks and Coordination Polymers as Alternative Phosphors for Energy Efficient Lighting Devices,” Coordination Chemistry Reviews 373 (2018): 116–147.

[22] X.‐Y. Liu , W. P. Lustig , and J. Li , “Functionalizing Luminescent Metal–Organic Frameworks for Enhanced Photoluminescence,” ACS Energy Letters 5 (2020): 2671–2680.

[23] S. Mendiratta , M. Usman , T.‐T. Luo , et al., “Anion‐Controlled Dielectric Behavior of Homochiral Tryptophan‐Based Metal–Organic Frameworks,” Crystal Growth and Design 14 (2014): 1572–1579.

[24] S. Mendiratta , M. Usman , T. T. Luo , S. F. Lee , Y. C. Lin , and K. L. Lu , “Guest Dependent Dielectric Properties of Nickel (II)‐Based Supramolecular Networks,” CrystEngComm 16 (2014): 6309–6315.

[25] S. Mendiratta , M. Usman , T.‐W. Tseng , et al., “Low Dielectric Behavior of a Robust, Guest‐Free Magnesium (II)–Organic Framework: A Potential Application of an Alkaline‐Earth Metal Compound,” European Journal of Inorganic Chemistry 2015 (2015):1669–1674.

[26] S. Mendiratta , M. Usman , C.‐C. Chang , et al., “Zn(II)‐Based Metal–organic Framework: an Exceptionally Thermally Stable, Guest‐Free Low Dielectric Material,” Journal of Materials Chemistry C 5 (2017): 1508–1513.

[27] S. Eslava , L. Zhang , S. Esconjauregui , et al., “Metal‐Organic Framework ZIF‐8 Films As Low‐κ Dielectrics in Microelectronics,” Chemistry of Materials 25 (2013): 27–33.

[28] P. Yang , X. He , M.‐X. Li , et al., “The First Homochiral Coordination Polymer with Temperature‐Independent Piezoelectric and Dielectric Properties,” Journal of Materials Chemistry 22 (2012): 2398–2400.

[29] F. Wang , C.‐Y. Ni , Q. Liu , et al., “[Pb(Tab)2(4,4′‐Bipy)](PF_6_)_2_: Two‐Step Ambient Temperature Quantitative Solid‐State Synthesis, Structure and Dielectric Properties,” Chemical Communications 49 (2013): 9248–9250. 23945650 10.1039/c3cc45066j

[30] S. Galli , A. Cimino , J. F. Ivy , et al., “Fluorous Metal–Organic Frameworks and Nonporous Coordination Polymers as Low‐κ Dielectrics,” Advanced Functional Materials 29 (2019): 1904707.

[31] M. Krishtab , I. Stassen , T. Stassin , et al., “Vapor‐Deposited Zeolitic Imidazolate Frameworks as Gap‐Filling Ultra‐Low‐k Dielectrics,” Nature Communications 10 (2019): 3729. 10.1038/s41467-019-11703-x PMC6700180 31427584

[32] W. Xu , S. S. Yu , H. Zhang , and H. B. Duan , “A Three‐Dimensional Metal–organic Framework for a Guest‐Free Ultra‐Low Dielectric Material,” RSC Advances 9 (2019): 16183–16186. 35521392 10.1039/c9ra03032h PMC9064416

[33] D.‐L. Zhang , Q.‐K. Feng , S.‐L. Zhong , D.‐F. Liu , Y. Zhao , and Z.‐M. Dang , “Tunable Dielectric Performance of Porphyrin Based Metal−organic Frameworks with Polar Molecule Confinement,” Composites Communications 25 (2021): 100734.

[34] A. S. Babal , B. E. Souza , A. F. Möslein , M. Gutiérrez , M. D. Frogley , and J.‐C. Tan , “Broadband Dielectric Behavior of an MIL‐100 Metal–Organic Framework as a Function of Structural Amorphization,” ACS Applied Electronic Materials 3 (2021): 1191–1198.

[35] A. S. Babal , L. Donà , M. R. Ryder , et al., “Impact of Pressure and Temperature on the Broadband Dielectric Response of the HKUST‐1 Metal–Organic Framework,” The Journal of Physical Chemistry C 123 (2019): 29427–29435.

[36] A. S. Babal and J.‐C. Tan , “Influence of Mechanical, Thermal, and Electrical Perturbations on the Dielectric Behaviour of Guest‐Encapsulated HKUST‐1 Crystals,” Journal of Materials Chemistry C 8 (2020): 12886–12892.

[37] A. Pathak , G. R. Chiou , N. R. Gade , et al., “High‐κ Samarium‐Based Metal–Organic Framework for Gate Dielectric Applications,” ACS Applied Materials and Interfaces 9 (2017): 21872−21878. 28594158 10.1021/acsami.7b03959

[38] R. Singh and R. K. Ulrich , “High and Low Dielectric Constant Materials,” The Electrochemical Society Interface 8 (1999): 26–30.

[39] M. Usman , P.‐H. Feng , K.‐R. Chiou , et al., “Polar Molecule Confinement Effects on Dielectric Modulations of Sr‐Based Metal–Organic Frameworks,” ACS Applied Electronic Materials 1 (2019): 836–844.

[40] S. Kamal , K. R. Chiou , S. Batjargal , et al., “Thermally Stable Indium Based Metal–organic Frameworks with High Dielectric Permittivity,” Journal of Materials Chemistry C 8 (2020): 9724–9733.

[41] S. Kamal , A. I. Inamdar , K. R. Chiou , et al., “Functional Groups Assisted Tunable Dielectric Permittivity of Guest‐Free Zn‐Based Coordination Polymers for Gate Dielectrics,” Chemistry – A European Journal 28 (2022): e202103905. 35318746 10.1002/chem.202103905

[42] Q. Ye , Y.‐M. Song , G.‐X. Wang , et al., “Ferroelectric Metal−Organic Framework with a High Dielectric Constant,” Journal of the American Chemical Society 128 (2006): 6554–6555. 16704244 10.1021/ja060856p

[43] T. Hang , D.‐W. Fu , Q. Ye , H.‐Y. Ye , R.‐G. Xiong , and S. D. Huang , “Tanklike Metal−Organic Framework Filled with Perchloric Acid and Its Dielectric−Ferroelectric Properties,” Crystal Growth and Design 9 (2009): 2054–2056.

[44] P.‐C. Guo , Z. Chu , X.‐M. Ren , W.‐H. Ning , and W. Jin , “Comparative Study of Structures, Thermal Stabilities and Dielectric Properties for a Ferroelectric MOF [Sr(μ‐BDC)(DMF)]_∞_ with Its Solvent‐Free Framework,” Dalton Transactions 42 (2013): 6603–6610. 23478356 10.1039/c3dt32880e

[45] Q. Chen , P.‐C. Guo , S.‐P. Zhao , J.‐L. Liu , and X.‐M. Ren , “A Rhombus Channel Metal‐Organic Framework Comprised of Sr^2+^ and Thiophene‐2, 5‐Dicarboxylic Acid Exhibiting Novel Dielectric Bistability,” CrystEngComm 15 (2013): 1264–1270.

[46] P. C. Guo , T. Y. Chen , X. M. Ren , W. H. Ning , and W. Jin , “A Low‐κ Dielectric Metal‐Organic‐Framework Compound Showing Novel Three‐Step Dielectric Relaxations Originating from Orientational Motion of Dipolar Guest Molecules,” New Journal of Chemistry 38 (2014): 2254–2257.

[47] M. Mączka , A. Ciupa , A. Gągor , et al., “Perovskite Metal Formate Framework of [NH_2_‐CH^+^ ‐NH_2_]Mn(HCOO)_3_]: Phase Transition, Magnetic, Dielectric, and Phonon Properties,” Inorganic Chemistry 53 (2014): 5260–5268. 24785192 10.1021/ic500479e

[48] R. Shang , Z.‐M. Wang , and S. Gao , “A 36‐Fold Multiple Unit Cell and Switchable Anisotropic Dielectric Responses in an Ammonium Magnesium Formate Framework,” Angewandte Chemie International Edition 54 (2015): 2534–2537. 25585529 10.1002/anie.201411005

[49] B.‐T. Qu , J.‐C. Lai , S. Liu , F. Liu , Y.‐D. Gao , and X.‐Z. You , “Cu‐ and Ag‐Based Metal–Organic Frameworks with 4‐Pyranone‐2,6‐Dicarboxylic Acid: Syntheses, Crystal Structures, and Dielectric Properties,” Crystal Growth and Design 15 (2015): 1707–1713.

[50] W.‐J. Li , J. Liu , Z.‐H. Sun , et al., “Integration of Metal‐Organic Frameworks into an Electrochemical Dielectric Thin Film for Electronic Applications,” Nature Communications 7 (2016): 11830. 10.1038/ncomms11830 PMC4906389 27282348

[51] B. Banday , V. Kumar , S. Murugavel , and A. Ramanan , “Strontium‐Carboxylate‐Based Coordination Polymers: Synthesis, Structure and Dielectric Properties,” ChemistrySelect 4 (2019): 4756–4766.

[52] B. Balendra , A. Banday , S. Murugavel , P. K. Kanaujia , G. V. Prakash , and A. Ramanan , “Calcium and Strontium Coordination Polymers Based on Rigid and Flexible Aromatic Dicarboxylates: Synthesis, Structure, Photoluminescence and Dielectric Properties,” ChemistrySelect 2 (2017): 8567–8576.

[53] T. Rom , N. Kumar , M. Sharma , A. Gaur , and A. K. Paul , “Colossal Dielectric Responses from the Wide Band Gap 2D‐Semiconducting Amine Templated Hybrid Framework Materials,” Inorganic Chemistry 59 (2020): 9465–9470. 32584035 10.1021/acs.inorgchem.0c01239

[54] J.‐H. Dou , L. Sun , Y. Ge , et al., “Signature of Metallic Behavior in the Metal–Organic Frameworks M_3_ (hexaiminobenzene)_2_ (M = Ni, Cu),” Journal of the American Chemical Society 139 (2017): 13608–13611. 28910095 10.1021/jacs.7b07234

[55] M. Usman , S. Mendiratta , S. Batjargal , et al., “Semiconductor Behavior of a Three‐Dimensional Strontium‐Based Metal–Organic Framework,” ACS Applied Materials and Interfaces 7 (2015): 22767–22774. 26414295 10.1021/acsami.5b07228

[56] A. M. Smith and S. Nie , “Semiconductor Nanocrystals: Structure, Properties, and Band Gap Engineering,” Accounts of Chemical Research 43 (2010): 190–200. 19827808 10.1021/ar9001069 PMC2858563

[57] M. Usman , K. P. Bera , G. Haider , et al., “Single‐Molecule‐Based Electroluminescent Device as Future White Light Source,” ACS Applied Materials and Interfaces 11 (2019): 4084–4092. 30604616 10.1021/acsami.8b17107

[58] T. W. Tseng , T. T. Luo , S. H. Liao , K. H. Lu , and K.‐L. Lu , “Isorecticular Synthesis of Dissectible Molecular Bamboo Tubes of Hexarhenium(I) Benzene‐1,2,3,4,5,6‐hexaolate Complexes,” Angewandte Chemie International Edition 128 (2016): 8483–8487. 10.1002/anie.201602327 27126190

[59] A. I. Inamdar , B. Sainbileg , C.‐J. Lin , et al., “Regimented Charge Transport Phenomena in Semiconductive Self‐Assembled Rhenium Nanotubes,” ACS Applied Materials and Interfaces 14 (2022): 12423–12433. 35254046 10.1021/acsami.2c00665

[60] A. I. Inamdar , H. K. Bangolla , K.‐F. Ho , et al., “Band Gap Engineering, Dark Conductivity, and Photoconductive Properties of Semiconductive Self‐Assembled Rhenium Nanotube by Structural Topology Modification,” ACS Applied Electronic Materials 7 (2025): 533–541.

[61] S. Kamal , A. I. Inamdar , K. R. Chiou , et al., “Semiconducting Paddle‐Wheel Metal–Organic Complex with a Compact Cu–S Cage,” The Journal of Physical Chemistry C 126 (2022): 6300–6307.

[62] J.‐Y. Wang , W.‐H. Li , Z. Wei , et al., “A Hydrophobic Semiconducting Metal–organic Framework Assembled from Silver Chalcogenide Wires,” Chemical Communications 56 (2020): 2091–2094. 31960846 10.1039/c9cc08402a

[63] A. A. Talin , A. Centrone , A. C. Ford , et al., “Tunable Electrical Conductivity in Metal‐Organic Framework Thin‐Film Devices,” Science 343 (2014): 66–69. 24310609 10.1126/science.1246738

[64] Z. Guo , D. K. Panda , K. Maity , et al., “Modulating the Electrical Conductivity of Metal–organic Framework Films with Intercalated Guest π‐Systems,” Journal of Materials Chemistry C 4 (2016): 894–899.

[65] Q.‐X. Wang and C.‐Y. Zhang , “Oriented Synthesis of One‐Dimensional Polypyrrole Molecule Chains in a Metal‐Organic Framework,” Macromolecular Rapid Communications 32 (2011): 1610–1614. 21732469 10.1002/marc.201100305

[66] S. Goswami , D. Ray , K. Otake , et al., “A Porous, Electrically Conductive Hexa‐Zirconium( Iv) Metal–organic Framework,” Chemical Science 9 (2018): 4477–4482. 29896389 10.1039/c8sc00961a PMC5956983

[67] G. Skorupskii , B. A. Trump , T. W. Kasel , C. M. Brown , C. H. Hendon , and M. Dincă , “Efficient and Tunable One‐Dimensional Charge Transport in Layered Lanthanide Metal–organic Frameworks,” Nature Chemistry 12 (2020): 131–136. 10.1038/s41557-019-0372-0 PMC11060427 31767997

[68] T. L. A. Nguyen , R. Demir‐Cakan , T. Devic , et al., “3‐D Coordination Polymers Based on the Tetrathiafulvalenetetracarboxylate (TTF‐TC) Derivative: Synthesis, Characterization, and Oxidation Issues,” Inorganic Chemistry 49 (2010): 7135–7143. 20597467 10.1021/ic100950n

[69] S. S. Park , E. R. Hontz , L. Sun , et al., “Cation‐Dependent Intrinsic Electrical Conductivity in Isostructural Tetrathiafulvalene‐Based Microporous Metal–Organic Frameworks,” Journal of the American Chemical Society 137 (2015): 1774–1777. 25597934 10.1021/ja512437u

[70] S. Takaishi , M. Hosoda , T. Kajiwara , et al., “Electroconductive Porous Coordination Polymer Cu[Cu(pdt)_2_] Composed of Donor and Acceptor Building Units,” Inorganic Chemistry 48 (2009): 9048–9050. 19067544 10.1021/ic802117q

[71] C. Jia , D. Zhang , C.‐M. Liu , W. Xu , H. Hu , and D. Zhu , “Novel Silver (I) Complexes Derived from Tetrakis (methylthio) Tetrathiafulvalene and Bis (ethylenedithio) Tetrathiafulvalene with 3D and 1D Structures,” New Journal of Chemistry 26 (2002): 490–494.

[72] K. Thurmer , C. Schneider , V. Stavila , et al., “Surface Morphology and Electrical Properties of Cu_3_ BTC_2_ Thin Films Before and After Reaction with TCNQ,” ACS Applied Materials and Interfaces 10 (2018): 39400–39410. 30354047 10.1021/acsami.8b15158

[73] X. Huang , P. Sheng , Z. Tu , et al., “A Two‐Dimensional π–d Conjugated Coordination Polymer with Extremely High Electrical Conductivity and Ambipolar Transport Behaviour,” Nature Communications 6 (2015): 7408. 10.1038/ncomms8408 PMC4490364 26074272

[74] Z. Jin , J. Yan , X. Huang , et al., “Solution‐Processed Transparent Coordination Polymer Electrode for Photovoltaic Solar Cells,” Nano Energy 40 (2017): 376–381.

[75] X. Huang , H. Li , Z. Tu , et al., “Highly Conducting Neutral Coordination Polymer with Infinite Two‐Dimensional Silver–Sulfur Networks,” Journal of the American Chemical Society 140 (2018): 15153–15156. 30207157 10.1021/jacs.8b07921

[76] R. Dong , Z. Zhang , D. C. Tranca , et al., “A Coronene‐Based Semiconducting Two‐Dimensional Metal‐Organic Framework with Ferromagnetic Behavior,” Nature Communications 9 (2018): 2637. 10.1038/s41467-018-05141-4 PMC6035257 29980687

[77] M.‐S. Yao , X.‐J. Lv , Z.‐H. Fu , et al., “Layer‐by‐Layer Assembled Conductive Metal–Organic Framework Nanofilms for Room‐Temperature Chemiresistive Sensing,” Angewandte Chemie International Edition 56 (2017): 16510–16514. 29071780 10.1002/anie.201709558

[78] Y. Cui , J. Yan , Z. Chen , et al., “[Cu_3_(C_6_Se_6_)]n: The First Highly Conductive 2D π–d Conjugated Coordination Polymer Based on Benzenehexaselenolate,” Advanced Science 6 (2019): 1802235. 31065526 10.1002/advs.201802235 PMC6498113

[79] H. Jia , Y. Yao , J. Zhao , Y. Gao , Z. Luo , and P. Du , “A Novel Two‐Dimensional Nickel Phthalocyanine‐Based Metal–organic Framework for Highly Efficient Water Oxidation Catalysis,” Journal of Materials Chemistry A 6 (2018): 1188–1195.

[80] M. Usman , G. Haider , S. Mendiratta , T.‐T. Luo , Y.‐F. Chen , and K.‐L. Lu , “Continuous Broadband Emission from a Metal–organic Framework as a Human‐Friendly White Light Source,” Journal of Materials Chemistry C 4 (2016): 4728–4732.

[81] K. P. Bera , G. Haider , M. Usman , et al., “Trapped Photons Induced Ultrahigh External Quantum Efficiency and Photoresponsivity in Hybrid Graphene/Metal‐Organic Framework Broadband Wearable Photodetectors,” Advanced Functional Materials 28 (2018): 1804802.

[82] K. P. Bera , S. Kamal , A. I. Inamdar , et al., “Intrinsic Ultralow‐Threshold Laser Action from Rationally Molecular Design of Metal–Organic Framework Materials,” ACS Applied Materials and Interfaces 12 (2020): 36485–36495. 32678568 10.1021/acsami.0c07890

[83] S. Kamal , K. P. Bera , M. Usman , et al., “Phosphor‐Free Electrically Driven White Light Emission from Nanometer‐Thick Barium–Organic Framework Films,” ACS Applied Nano Materials 4 (2021): 2395–2403.

[84] Q. Yang , W. Wang , Y. Yang , et al., “Pressure Treatment Enables White‐Light Emission in Zn‐IPA MOF via Asymmetrical Metal‐Ligand Chelate Coordination,” Nature Communications 16 (2025): 696. 10.1038/s41467-025-55978-9 PMC11736072 39814792

[85] X.‐G. Yang , Y.‐J. Chen , P.‐P. Yin , et al., “Low Thermal Quenching of Metal Halide‐Based Metal–organic Framework Phosphor for Light‐Emitting Diodes,” Chemical Science 15 (2024): 14202–14208. 10.1039/d4sc04228j PMC11322981 39149214

[86] T. Mondal , S. Mondal , S. Bose , D. Sengupta , U. K. Ghorai , and S. K. Saha , “Pure White Light Emission from a Rare Earth‐Free Intrinsic Metal–organic Framework and Its Application in a WLED,” Journal of Materials Chemistry C 6 (2018): 614–621.

[87] M.‐S. Wang , S.‐P. Guo , Y. Li , et al., “A Direct White‐Light‐Emitting Metal−Organic Framework with Tunable Yellow‐to‐White Photoluminescence by Variation of Excitation Light,” Journal of the American Chemical Society 131 (2009): 13572–13573. 19772357 10.1021/ja903947b

[88] C. Peng , X. Song , J. Yin , G. Zhang , and H. Fei , “Intrinsic White‐Light‐Emitting Metal–Organic Frameworks with Structurally Deformable Secondary Building Units,” Angewandte Chemie International Edition 58 (2019): 7818–7822. 30957350 10.1002/anie.201903665

[89] X. Yang and D. Yan , “Direct White‐Light‐Emitting and Near‐Infrared Phosphorescence of Zeolitic Imidazolate Framework‐8,” Chemical Communications 53 (2017): 1801–1804. 28098276 10.1039/c6cc09706e

[90] C. Wang , Z. Yin , W.‐M. Ma , et al., “Near Sunlight Continuous Broadband White‐Light Emission by Single‐Phase Zn(II)‐1,3,5‐Benzenetricarboxylate MOFs,” Dalton Transactions 48 (2019): 14966–14970. 31552978 10.1039/c9dt03388b

[91] Z. Yin , W.‐M. Ma , C. Wang , et al., “Color‐Tuning and Near‐Sunlight White Emission in Highly Stable Rod‐Spacer MOFs with Defective Dicubane Based Lead(II)‐Carboxyl Chains,” Inorganic Chemistry 58 (2019): 16171–16179. 31718168 10.1021/acs.inorgchem.9b02697

[92] Y. Tang , T. Xia , T. Song , Y. Cui , Y. Yang , and G. Qian , “Efficient Energy Transfer Within Dyes Encapsulated Metal–Organic Frameworks to Achieve High Performance White Light‐Emitting Diodes,” Advanced Optical Materials 6 (2018): 1800968.

[93] X. Wang , Z. Li , W. Ying , et al., “Blue Metal–organic Framework Encapsulated Denatured R‐Phycoerythrin Proteins for a White‐Light‐Emitting Thin Film,” Journal of Materials Chemistry C 8 (2020): 240–246.

[94] Y. P. Xia , C. X. Wang , L. C. An , et al., “Utilizing an Effective Framework to Dye Energy Transfer in a Carbazole‐Based Metal–organic Framework for High Performance White Light Emission Tuning,” Inorganic Chemistry Frontiers 5 (2018): 2868–2874.

[95] T. Mondal , S. Bose , A. Husain , U. K. Ghorai , and S. K. Saha , “White Light Emission from Single Dye Incorporated Metal Organic Framework,” Optical Materials 100 (2020): 109706.

[96] A. Wang , Y. L. Hou , F. Kang , et al., “Rare Earth‐Free Composites of Carbon Dots/Metal–organic Frameworks as White Light Emitting Phosphors,” Journal of Materials Chemistry C 7 (2019): 2207–2211.

[97] C. Y. Sun , X. L. Wang , X. Zhang , et al., “Efficient and Tunable White‐Light Emission of Metal–organic Frameworks by Iridium‐Complex Encapsulation,” Nature Communications 4 (2013): 2717. 10.1038/ncomms3717 PMC3831296 24212250

[98] T. Mondal , D. Haldar , A. Ghosh , U. K. Ghorai , and S. K. Saha , “A MOF Functionalized with CdTe Quantum Dots as an Efficient White Light Emitting Phosphor Material for Applications in Displays,” New Journal of Chemistry 44 (2020): 55–63.

[99] Y. Cui , T. Song , J. Yu , Y. Yang , Z. Wang , and G. Qian , “Dye Encapsulated Metal‐Organic Framework for Warm‐White LED with High Color‐Rendering Index,” Advanced Functional Materials 25 (2015): 4796–4802.

[100] W. Xie , W.‐W. He , D.‐Y. Du , et al., “A Stable Alq3@MOF Composite for White‐Light Emission,” Chemical Communications 52 (2016): 3288–3291. 26732031 10.1039/c5cc08703a

[101] J. Othong , J. Boonmak , V. Promarak , F. Kielar , and S. Youngme , “Sonochemical Synthesis of Carbon Dots/Lanthanoid MOFs Hybrids for White Light‐Emitting Diodes with High Color Rendering,” ACS Applied Materials and Interfaces 11 (2019): 44421–44429. 31674176 10.1021/acsami.9b13814

[102] D. F. Sava , L. E. S. Rohwer , M. A. Rodriguez , and T. M. Nenoff , “Intrinsic Broad‐Band White‐Light Emission by a Tuned, Corrugated Metal–Organic Framework,” Journal of the American Chemical Society 134 (2012): 3983–3986. 22339608 10.1021/ja211230p

[103] B. H. Wang and B. Yan , “Tunable Multi‐Color Luminescence and White Emission in Lanthanide Ion Functionalized Polyoxometalate‐Based Metal–organic Frameworks Hybrids and Fabricated Thin Films,” Journal of Alloys and Compounds 777 (2019): 415–422.

[104] J. X. Li , Q. L. Guan , Y. Wang , et al., “A Lanthanide–Organic Crystalline Framework Material Encapsulating 1,3,6,8‐tetrakis (p‐Benzoic Acid) Pyrene: Selective Sensing of Fe^3+^, Cr_2_O_7_ ^2−^ and Colchicine and White‐Light Emission,” New Journal of Chemistry 44 (2020): 1446–1454.

[105] X. Y. Liu , K. Xing , Y. Li , C. K. Tsung , and J. Li , “Three Models to Encapsulate Multicomponent Dyes into Nanocrystal Pores: A New Strategy for Generating High‐Quality White Light,” Journal of the American Chemical Society 141 (2019): 14807–14813. 31424923 10.1021/jacs.9b07236

[106] Y. H. Luo , A. D. Xie , W. C. Chen , et al., “Multifunctional Anionic Indium–organic Frameworks for Organic Dye Separation, White‐Light Emission and Dual‐Emitting Fe^3+^ Sensing,” Journal of Materials Chemistry C 7 (2019): 14897–14903.

